# Harnessing Neuroplasticity to Promote Brain Health in Aging Adults: Protocol for the MOVE-Cog Intervention Study

**DOI:** 10.2196/33589

**Published:** 2021-11-23

**Authors:** Danylo F Cabral, Carrie A Hinchman, Christina Nunez, Jordyn Rice, David A Loewenstein, Lawrence P Cahalin, Tatjana Rundek, Alvaro Pascual-Leone, Joyce Gomes-Osman

**Affiliations:** 1 Department of Physical Therapy University of Miami Miller School of Medicine Coral Gables, FL United States; 2 School of Medicine New York Medical College Valhalla, NY United States; 3 Center for Cognitive Neuroscience and Aging University of Miami Miller School of Medicine Miami, FL United States; 4 Department of Neurology University of Miami Miller School of Medicine Miami, FL United States; 5 Evelyn McKnight Brain Institute University of Miami Miller School of Medicine Miami, FL United States; 6 Department of Neurology Harvard Medical School Boston, MA United States; 7 Hinda and Arthur Marcus Institute for Aging Research Hebrew SeniorLife Rosindale, MA United States; 8 Deanna and Sidney Wolk Center for Memory Health Hebrew SeniorLife Rosindale, MA United States; 9 Guttmann Brain Health Institute Barcelona Spain

**Keywords:** exercise, neuroplasticity, cognition, brain health, cardiorespiratory fitness, cardiovascular function, trophic factors, telehealth, aging adult

## Abstract

**Background:**

Extensive evidence supports a link between aerobic exercise and cognitive improvements in aging adults. A major limitation with existing research is the high variability in cognitive response to exercise. Our incomplete understanding of the mechanisms that influence this variability and the low adherence to exercise are critical knowledge gaps and major barriers for the systematic implementation of exercise for promoting cognitive health in aging.

**Objective:**

We aimed to provide an in-person and remotely delivered intervention study protocol with the main goal of informing the knowledge gap on the mechanistic action of exercise on the brain by characterizing important mechanisms of neuroplasticity, cardiorespiratory fitness response, and genetics proposed to underlie cognitive response to exercise.

**Methods:**

This is an open-label, 2-month, interventional study protocol in neurologically healthy sedentary adults. This study was delivered fully in-person and in remote options. Participants underwent a total of 30 sessions, including the screening session, 3 pretest (baseline) assessments, 24 moderate-to-vigorous aerobic exercise sessions, and 3 posttest assessments. We recruited participants aged 55 years and above, sedentary, and cognitively healthy. Primary outcomes were neuroplasticity, cognitive function, and cardiorespiratory fitness. Secondary outcomes included genetic factors, endothelium function, functional mobility and postural control, exercise questionnaires, depression, and sleep. We also explored study feasibility, exercise adherence, technology adaptability, and compliance of both in-person and remote protocols.

**Results:**

The recruitment phase and data collection of this study have concluded. Results are expected to be published by the end of 2021 or in early 2022.

**Conclusions:**

The data generated in these studies will introduce tangible parameters to guide the development of personalized exercise prescription models for maximal cognitive benefit in aging adults. Successful completion of the specific aims will enable researchers to acquire the appropriate expertise to design and conduct studies by testing personalized exercise interventions in person and remotely delivered, likely to be more effective at promoting cognitive health in aging adults.

**Trial Registration:**

ClinicalTrials.gov NCT03804528; http://clinicaltrials.gov/ct2/show/NCT03804528

**International Registered Report Identifier (IRRID):**

RR1-10.2196/33589

## Introduction

### Cognitive Aging

Individuals aged 65 years and older will compose 30% of the global population over the next 30 years, totaling approximately 2 billion people [1]. This increase in human longevity has unfortunately not been accompanied by a comparable increase in healthy longevity (health span). Age-related cognitive decline impacts healthy longevity by leading to substantial disability and decreased quality of life. Cognitive health is critical for healthy longevity because it is highly linked to one’s functional independence in managing important aspects of life such as living independently in one’s home and managing health care and finances. Pharmacological approaches to treating age-related cognitive decline in midlife to later life have been limited [2]. In contrast, interventions that increase levels of physical activity have been effective in improving cognitive function in aging adults [3-5]. But there is considerable interindividual variability in cognitive outcomes after exercise. Greater understanding of this variability and the underpinning mechanisms are necessary to improve the effectiveness of exercise in prevention and remediation of age-related cognitive decline.

### Variability in Cognitive Response to Exercise

Extensive evidence supports a link between aerobic exercise and cognitive improvements in aging adults. Heterogeneity in exercise-induced cognitive improvements remains the most significant barrier to achieving optimal efficacy of exercise for brain health in aging. Some individuals show robust cognitive benefits from exercise, while others show less pronounced improvements. To answer this question, measures that capture the effects of exercise at the brain level and knowledge of factors explaining the differences in cognitive response to exercise are needed. For example, the optimal and necessary exercise dose and regimen required to achieve cognitive benefits are unknown. Likewise, it is unclear which cognitive domains show most reliable and consistent improvements following exercise. Similarly, it is unclear which neuropsychological outcomes are most sensitive to capture exercise-induced cognitive gains. Advancing understanding of differences in cognitive response to exercise requires concurrent examination of associated neuroplasticity and cardiovascular changes after exercise. Aerobic exercise, with its cardiorespiratory gains, contributes to cognition by optimizing cerebrovascular function, ultimately leading to more efficient neural processing [6]. At the brain level, neuroplasticity is an important mechanism and driver of aerobic exercise–induced cognitive improvements.

### Evaluating Mechanisms of Neuroplasticity to Capture the Effects of Exercise in the Brain

Neuroplasticity is broadly defined as a change in neural structure and function in response to experience or environmental stimuli. A critical component of neuroplasticity in response to exercise is long-lasting synaptic potentiation. Direct electrical recordings of hippocampal neurons show synaptic enhancement (ie, long-term potentiation) [7] after exercise that correlates with improved cognitive outcomes [8-14]. Prior studies using transcranial magnetic stimulation (TMS) demonstrated a substantial long-term potentiation–like neuroplasticity response to exercise that was relevant for exercise-induced cognitive gains [15]. Importantly, it is possible to assess neuroplasticity noninvasively and remotely by using transcranial alternating current stimulation (tACS) combined with electroencephalography (EEG). In this paradigm, small electrodes placed on the scalp deliver a short burst of tACS, which induces a controlled perturbation in the brain that is measured by EEG changes. The magnitude and duration of tACS-induced spectral EEG changes in EEG enable an assessment of neuroplastic mechanisms that is akin to the TMS/intermittent theta-burst stimulation (iTBS) approach [16,17]. tACS/EEG is uniquely suited for a home-based intervention as it can be delivered with a device designed for home use. Using established procedures via a secure video platform, the participant is instructed on how to appropriately place the EEG cap, and the study investigators conduct the assessment via remote neurophysiological monitoring. Thus, evaluation of tACS-induced spectral EEG changes is relevant to advance understanding of exercise-mediated cognitive benefits, with the important advantage of being suitable for home-based interventions.

### Low Adherence to Exercise Is a Major Obstacle

In addition to all mechanisms that influence the variability of cognitive gains in response to exercise, an important limitation of existing research is that adhering to supervised in-person exercise can be challenging for many aging adults. Given the increasing use of ubiquitous mobile technologies by aging adults, a remotely delivered exercise research program may facilitate recruitment and retention, address barriers to exercise participation, and accelerate the translation of our findings to a broader audience. This is especially relevant considering the post–COVID-19 pandemic and living in the new normal social practices.

Thus, our incomplete understanding of the mechanisms that influence this variability and low adherence to exercise are critical knowledge gaps and major barriers for the systematic implementation of exercise for promoting cognitive health in aging. The major goal of this study is to inform the knowledge gap on the mechanistic action of exercise on the brain by characterizing important mechanisms of neuroplasticity, cardiorespiratory fitness, mobility, endothelium function, and genetic factors proposed to underlie cognitive response to exercise. Addressing this gap with methods of clinical and translational research will aid the development of exercise interventions that can be individually tailored to establish maximal effectiveness.

### Specific Aims of the Study

Aim 1: Quantify improvements in cognitive performance after 8 weeks (150 minutes per week) of in-person and remotely delivered moderate-to-vigorous intensity aerobic exercise in sedentary aging adults aged 55 years and older. Hypothesis 1: We hypothesize improvements in overall cognitive performance after exercise and greater improvements in specific cognitive domains (eg, in executive function, as opposed to memory or language).

Aim 2: Quantify improvements in mechanisms of neuroplasticity and cardiorespiratory fitness response after 8 weeks (150 minutes per week) of in-person and remotely delivered moderate-to-vigorous intensity aerobic exercise in sedentary adults aged 55 years and older. Hypothesis 2: We hypothesize increased response in the mechanisms of neuroplasticity (as assessed by TMS-iTBS and tACS-induced spectral EEG changes) and increased cardiorespiratory response.

Aim 3: Determine an association between changes in neuroplasticity, cognitive performance, and cardiorespiratory fitness response after 8 weeks of moderate-to-vigorous intensity aerobic exercise in sedentary adults aged 55 years and older. Hypothesis 3: We hypothesize that a greater neuroplasticity response will be associated with greater improvement in cognitive performance and better cardiorespiratory response.

Aim 4: Determine whether postexercise cognitive gains, neuroplasticity, and cardiorespiratory response are moderated by baseline endothelial function and genetic factors. Hypothesis 4: Postexercise cognitive gains in neuroplasticity and cardiorespiratory response will be greater among individuals with greater endothelial function (brain-derived neurotrophic factors [BDNF] and vascular endothelial growth factor [VEGF]) and greater among individuals with an absence of apolipoprotein E e4 allele and Met BDNF allele.

### Exploratory Aims

Aim 5. To examine and compare the feasibility and adherence of an in-person and remotely delivered exercise trial consisting of 8 weeks of moderate-to-vigorous intensity aerobic exercise (150 minutes per week). Hypothesis 5: We predict that this study will be feasible, defined by successful recruitment of 40 participants in the in-person group and 40 participants in the remote group, and will demonstrate at least 80% adherence to the program.

Aim 6. To examine the adaptability and technology compliance of an in-person and remotely delivered exercise intervention and remote testing procedures, including neuroplasticity assessment, neurophysiological testing, cardiorespiratory fitness, and mobility in sedentary aging adults aged 55 years and older. Hypothesis 6: We hypothesize that it would be feasible to adapt and compare the exercise intervention and testing procedures and participants will have at least an 80% technology compliance.

## Methods

### Ethical Aspects and Study Design

This is an open label, 2-month, interventional study protocol in neurologically healthy sedentary adults. Participants underwent a total of 30 sessions, including the screening session, 3 pretest (baseline) assessments, 24 aerobic exercise sessions, and 3 posttest assessments. This study was conceptualized a priori to be delivered fully in-person. Due to challenges posed by the COVID-19 pandemic, social distancing practices, and aiming to create a feasible scenario to continue this study, we propose an additional option to safely adapt study methods to deliver the study in a home-based, fully remote manner. Importantly, this scenario also presented as an opportunity to collect meaningful data on our specific aims while translating this successful research program into a remote/home-based mode of delivery. A remote/home-based option may also yield valuable preliminary data relevant for planning future exercise studies in aging adults in the present new normal. Proposed changes included: (1) screening and additional inclusion criteria, (2) informed consent form and consenting procedures, (3) addition of a study kit, (4) remote testing procedures, (5) remote exercise intervention, and (6) additional safety plan.

All participants provided written informed consent prior to participation, and all forms and procedures were approved by the University of Miami institutional review board (IRB). This protocol and all protocol modifications were approved by the IRB.

**Figure 1:**
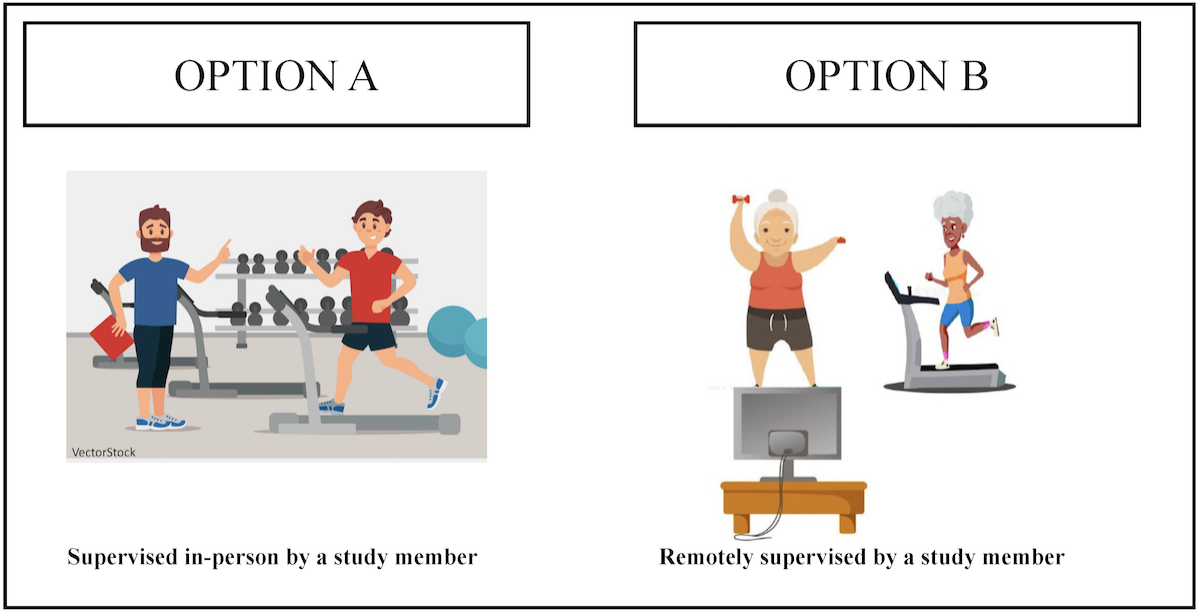
Study design options.

Therefore, this research project was offered using a 2-option design (Figure 1) consisting of the following:

- Option A: supervised in-person exercise intervention. This option was offered between February 2019 and March 2020
- Option B: home-based remotely supervised option of current methods to maximize recruitment. This option was offered between August 2020 and April 2021

### Recruitment and Consent

Participants were recruited using an open enrollment process. A total of 74 adults aged 55 years or older were recruited from August 2019 to April 2021. Participants for this study were recruited by advertising recruitment flyer on the Miller School of Medicine campus, University of Miami Coral Gables campus, and greater Miami community (eg, public libraries). In addition, an online research database tool (eg, ResearchMatch.org), the social media platform Nextdoor.com, and the University Research Informatics Data Environment were used as recruitment methods, and potential participants were identified and contacted via the Consent to Contact Initiative. Individuals who were interested in participating in the study were instructed to contact study staff via email or telephone to discuss the study details. At this time, the study was explained to the potential participants, who were screened to eligibility criteria. Participants who fulfilled preliminary eligibility criteria and showed further interest in participating were scheduled to proceed with a consent visit in-person or virtually via Zoom for Healthcare (Zoom Video Communications Inc) and Research Electronic Data Capture (REDCap) e-consent. During this meeting, individuals signed the consent form and followed the procedures for the study. Participants were compensated for their participation in the study.

### Eligibility Criteria

Participants were screened by collecting information to ensure their eligibility in the study. Inclusion criteria included individuals age 55 years and older, no clinically detectable cognitive impairment (Montreal Cognitive Assessment score ≥24), sedentary (defined as low category using the International Physical Activity Questionnaire–short form last 7 days), and English as primary language. An additional inclusion criterion for the remote group was basic computer skills (accessing an email or using the internet).

Exclusion criteria included any unstable medical condition (ie, uncontrolled hypertension or uncontrolled diabetes), medical contraindication to physical exercise, and contraindication to neuroplasticity assessment (TMS and tACS) [18]. No medication is an absolute exclusion from TMS and tACS. The decision to include a person in the study was made upon a review of the potential participant’s medical history, drug dose, history of recent medication changes or duration of treatment, vital signs sheet, physical ability readiness questionnaire, and a systems review performed by a licensed physical therapist who was member of the laboratory. Based on the results of this evaluation, if the participant was found to require further medical clearance, they were referred to their physician for further evaluation and assessment of potential eligibility in this study.

### Procedures

#### Study Timeline

**Figure 2:**
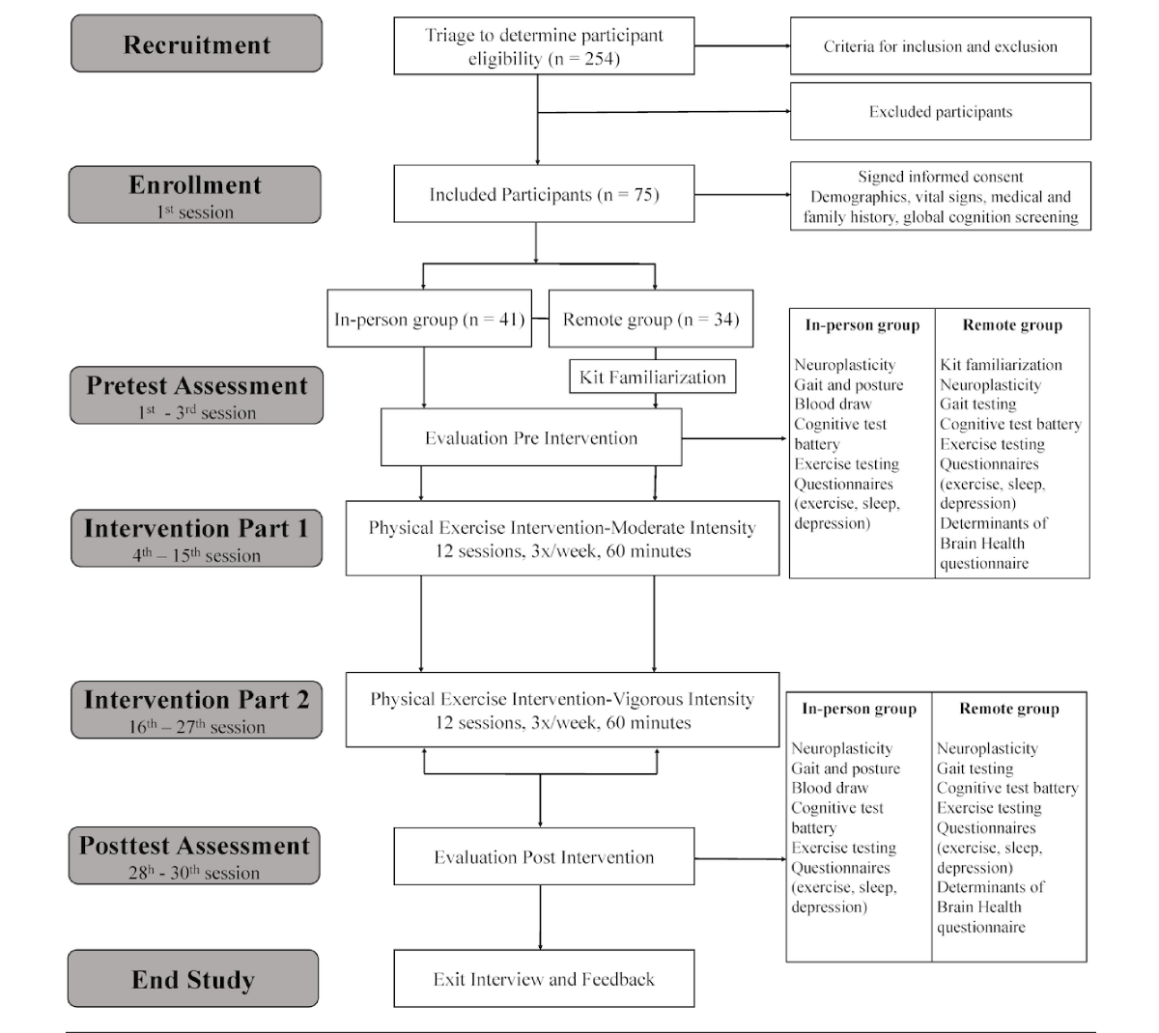
Study flowchart.

The total time for a participant to complete this study varied based on participant availability, but it was feasible to complete within 10 weeks (the intervention portion of the study was 8 weeks, with week 1 designated for pretesting and week 10 for posttesting). The following timeline was used to guide and track the intervention based on participant visit/session number and study delivery option (in-person vs remote) available as described in Figure 2.

#### In-Person Procedures

Screening was partially done during a phone interview but required 1 visit in person during week 1 to consent and collect physical measures (eg, vital signs), demographics, and medical and family history. All assessments were conducted by a study member who received specific training for each test. The assessments were divided into 3 different visits. Blood draw and neuroplasticity assessment were performed on the first visit, cognitive test battery and questionnaires were performed on the second visit, and gait and postural control assessment and exercise fitness testing were performed on the third visit. All testing procedures were conducted in the same order after the intervention with the same study member.

#### Remote Procedures

**Figure 3:**
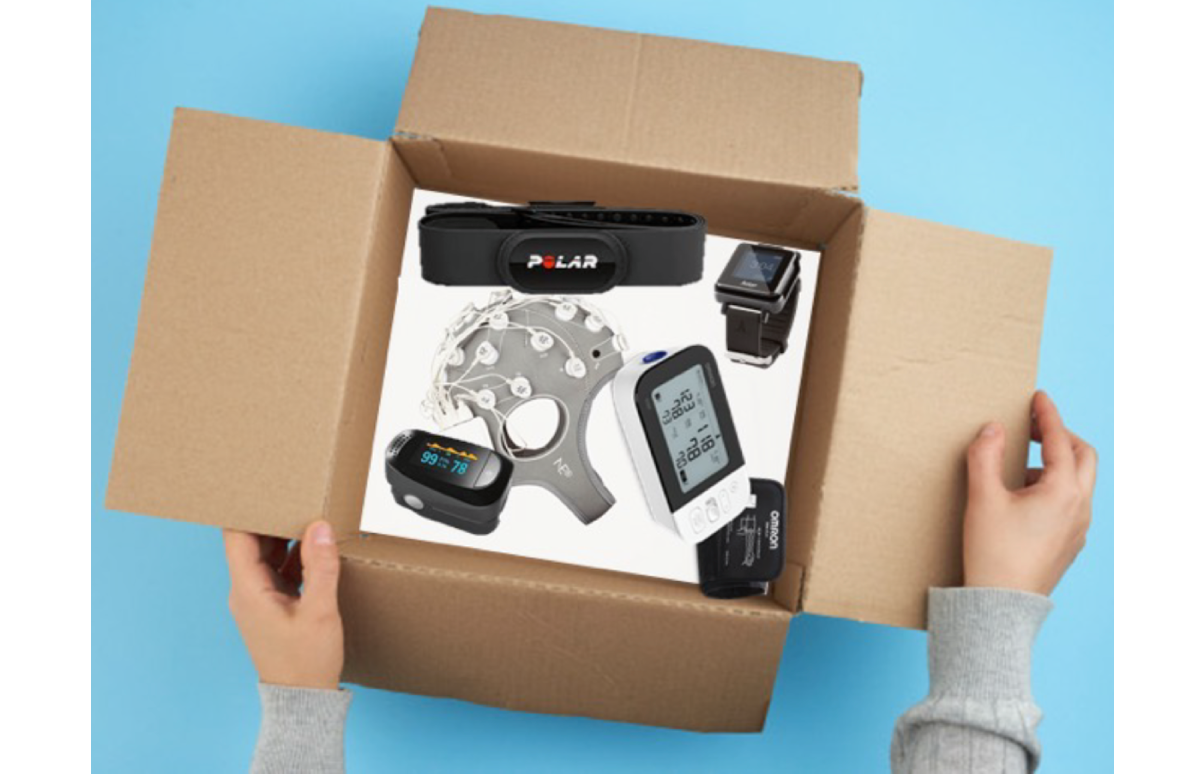
Items included in the study kit.

This option was an adaptation of the in-person methods that included an updating testing timeline to include a kit familiarization session. We updated our assessment to include outcome measures that were appropriate for remote delivery. We eliminated our genetic testing, as it was not possible to perform remotely. In this option, in-person visits were replaced with remote sessions. Upon signing informed consent to participate in the study, participants received a study kit delivered by a study member or through the mail (Figure 3) containing the devices that would be used during the pre- and postassessment tests and exercise intervention. The study kit included a (1) sphygmomanometer (OMRON BP7350, Omron Healthcare Inc); (2) heart rate monitor (Polar H10, Polar Electro Inc); (3) pulse oximeter (Diagnostix 2100 fingertip pulse oximeter, American Diagnostic Corp); (4) physical activity monitor (ActiGraph GT9X-BT Link, ActiGraph LLC); (5) tape measure, masking tape, and alcohol prep pads (isopropyl alcohol 70%); (6) noninvasive brain stimulation/EEG hybrid Starstim-Home (Neuroelectrics), including a tablet; (7) participant study booklet; and (8) prepaid shipping label to return the kit.

When the study kit arrived, before beginning any assessments or intervention, we scheduled a remote meeting using Zoom for Healthcare to familiarize the participant with all the tools in the study kit. The participant learned various Zoom platform functions; how to take their own blood pressure and oxygen saturation, put on the heart rate monitor and EEG cap, and set up the activity monitor; and proper safety and cleaning procedures. If needed, we scheduled multiple meetings to ensure the participant was properly familiarized with all the materials in the study kit. Participants also received a study booklet (Multimedia Appendix 1) containing specific recommendations on how to follow the study procedures, set up the study devices, and prepare for the assessment; definitions of important terms, and daily exercise sheets to be completed during the exercise sessions.

All assessments visits were monitored remotely using a secure, video platform through Zoom and were conducted by the study team members in the same order as the in-person study protocol.

### Intervention

#### In-Person Exercise Protocol

The physical exercise intervention was administered at the University of Miami Miller School of Medicine Wellness Center. Participants were given a time slot for the duration of the study, but flexibility with the intervention time frame was provided. Participants were supervised by a member of the study team (licensed physical therapists) at all times. Each participant engaged in 60-minute daily sessions delivered 3 times per week for 8 consecutive weeks (a total of 24 sessions).

**Figure 4:**
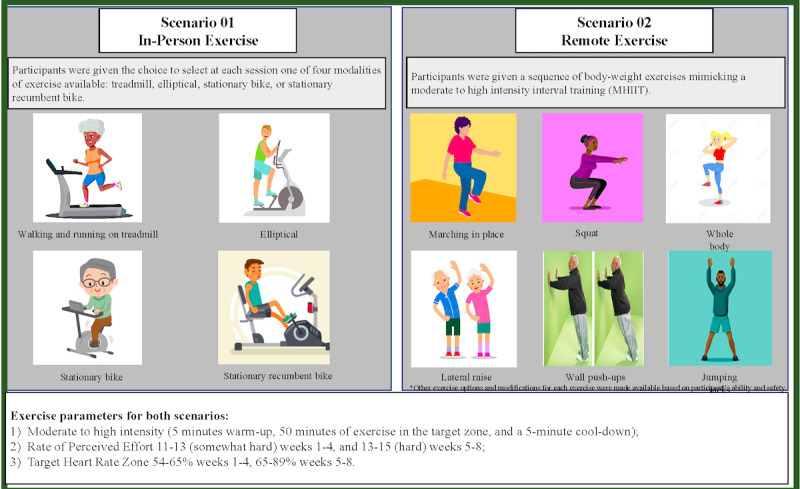
In-person and remote exercise protocol scenarios.

Participants could select 1 of 4 modalities of exercise available (treadmill, elliptical, stationary bike, or stationary recumbent bike) at each session. They were fitted with a heart rate monitor and were instructed to maintain 55% to 64% of maximal heart rate (determined by the Karvonen method) for the first 4 weeks of exercise and 65% to 89% of maximal heart rate for the second 4 weeks of exercise. During the sessions, heart rate and participant’s exerted effort (measured with the Borg scale) [19] were monitored prior to the session, every 5 minutes of the 50 minute session, and 5 minutes after the end of the session. The Borg scale is a numerical scale where 6 represents rest (no effort) and 20 represents maximal effort. Blood pressure was measured with participant seated for 5 minutes prior to the session and after completing the session. The session was discontinued if participant reported excessive discomfort (eg, musculoskeletal pain), and an appropriate referral was made as necessary. Participants were offered the opportunity to make up a missed session. Figure 4 shows a sample of exercise types for the in-person and remote protocols.

#### Remote Exercise Protocol

We have modified our 8-week, 3 per week in-person exercise intervention to be safely delivered as a home-based exercise program. Exercise sessions follow standard physical therapy protocols with existing safety procedures from the in-person sessions adapted to a virtual setting. Additional safety procedures included oxygen saturation measure, daily signs and symptoms questionnaire, daily geographical location, and daily emergency phone number. Home-based exercise sessions were monitored remotely by a physical therapist using Zoom for Healthcare. Participants were given the choice of exercise mode. Participants who had access to aerobic exercise equipment at home (treadmill, elliptical, or stationary bike) were able to use them in the same manner as scenario 1 (see Figure 4, scenario 1) with a remote investigator monitoring and instructing. Participants who did not have exercise equipment at home were given a routine of exercises that did not require any equipment and could be done safely in their homes. The body weight exercise program was developed by a group of 4 physical therapists formed by the principal investigator and study team members. Modifications for each exercise were tailored individually based on participant ability levels, available resources, and safety.

Exercises followed a standard format to ensure safety and adherence to study goals (Table 1). All participants completed a guided 5-minute warm-up. The intervention was structured in five 10-minute timed blocks consisting of 8 minutes of exercise and 2 minutes of rest. For the body weight protocol, participants were showed 2 exercises. They performed exercise A for 1 minute, exercise B for 1 minute, and then rested for 20 seconds. Participant then repeated the A-B sequence 3 more times for a total of 8 minutes of exercise and took an additional rest and water break for 1 minute to complete block 1. Participant completed 5 blocks using this format, completing a total of 10 unique exercises, and was then guided through a cool down where they slowly decreased exercise vigor to bring their heart rate down to baseline levels. Following exercise, the participant was asked to take their blood pressure, heart rate, and oxygen saturation and were monitored until they returned to within 20% of their baseline measurement. Each day of the week (eg, Monday, Wednesday, and Friday) had a different sequence of exercises.

The remote protocol followed the same intensity as the in-person protocol with an adaptation to mimic moderate-to-high intensity interval training (relative to their age-predicted maximal heart rate determined by the Karvonen method [heart rate (HR) = HRrest + ((intended fraction) * (HRmax – HRrest))], where HRmax = 220 – age, and intended fraction was 54% to 65% on weeks 1 to 4 and 65% to 90% on weeks 5 to 8). For participants taking β-blocker medication, an adjusted formula was used to estimate HRmax (HRmax = 119 + 0.5 [resting HR] − 0.5 [age]) [20]. We assessed perceived effort using the 20-point Borg scale. Table 1 shows the protocol of exercise for both groups.

**Table 1:** Remote exercise protocols.

Time	Steady state aerobic (personal equipment)	Body weight (no equipment)
0:00-4:59 (5 min)	Warm-up	Warm-up
5:00-14:59 (10 min) Work: 8 min, rest and water: 2 min	Block 1	Block 1: A/B
15:00-24:59 (10 min) Work: 8 min, rest and water: 2 min	Block 2	Block 2: C/D
25:00-34:59 (10 min) Work: 8 min, rest and water: 2 min	Block 3	Block 3: E/F
35:00-44:59 (10 min) Work: 8 min, rest and water: 2 min	Block 4	Block 4: G/H
45:00-54:59 (10 min) Work: 8 min, rest and water: 2 min	Block 5	Block 5: I/J
55:00-60:00 (5 min)	Cool down	Cool down

### Outcome Measures

All assessment measures were performed at baseline and after the 8-week exercise intervention. Table 2 shows the summary of assessments used in the in-person and remote groups.

**Table 2:** Outcomes and assessments.

Outcome measures			In-person assessment		Both		Remote assessment
**Primary**							
	**Neuroplasticity**						
		TMS/iTBS^a^		—^b^		tACS/EEG^c^	
	**Cognitive function**						
		—		Digit span subtest of the WAIS-4th^d^		—	
		—		Trail-Making Test Part B		—	
		—		RBANS^e^		—	
		—		DKEFS^f^		—	
		Stroop color-word test		—		TestMyBrain.org	
	**Cardiorespiratory fitness**						
		Incremental shuttle walking test		—		1-minute sit-to-stand test	
**Secondary**							
	**Functional mobility and postural control**						
		—		Timed Up-and-Go test (single and dual task)		—	
		Wireless accelerometer		—		—	
		90-second trials of walking		—		—	
		Standing postural control		—		—	
	**Blood collection and genetic testing**						
		Endothelium function (BDNF^g^, VEGF^h^, and CRP^i^ levels) and genetic factors (BDNF genes, and APOE^j^ e4 allele)		—		—	
	**Physical activity and exercise**						
		—		Lifetime Physical Activity questionnaire		—	
		—		Exercise Self-Efficacy, and Barriers and Motivators to Exercise		—	
		—		Physical Activity Self-Regulation Scale		—	
		—		—		ActiGraph GT9X Link	
	**Depression**						
		—		GDS^k^		—	
	**Sleep**						
		—		PSQI^l^		—	
		—		—		ActiGraph GT9X Link	
	**Brain health index**						
		—		—		BBHI^m^ determinants of brain health	
	**Study feasibility**						
		—		Exercise adherence and technology compliance		—	
		—		Exercise intensity adherence		—	
		—		Adaptability of the intervention and assessments methods		—	
	**Participant self-evaluation, study feedback, and plans for exercise**						
		Exit interview survey		—		Exit interview survey with additional questions	

^a^TMS/iTBS: transcranial magnetic stimulation/intermittent theta-burst stimulation.

^b^Not applicable.

^c^tACS/EEG: transcranial alternating current stimulation/electroencephalography.

^d^WAIS-4th: Wechsler Adult Intelligence Scale, 4th Edition.

^e^RBANS: Repeatable Neuropsychological Battery.

^f^DKEFS: Delis-Kaplan Executive Function System.

^g^BDNF: brain-derived neurotrophic factor.

^h^VEGF: vascular endothelial growth factor.

^i^CRP: C reactive protein.

^j^APOE: apolipoprotein E.

^k^GDS: Geriatric Depression Scale.

^l^PSQI: Pittsburgh Sleep Quality Index.

^m^BBHI: Barcelona Brain Health Institute.

### Primary Outcome Measures

#### Neuroplasticity Measures

##### TMS Plasticity

**Figure 5:**
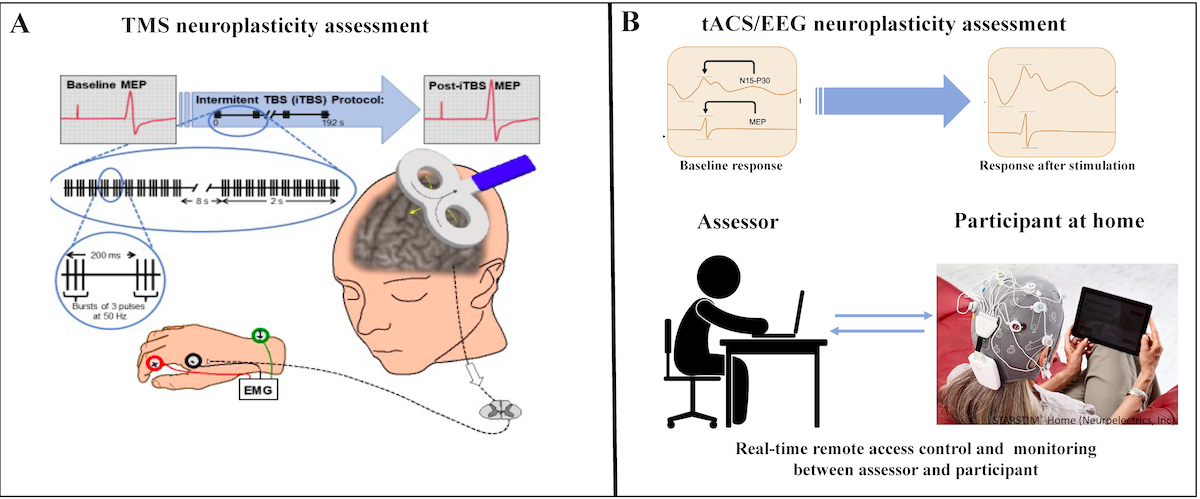
Experimental setup for transcranial magnetic stimulation (TMS) and transcranial alternating current stimulation/electroencephalography (tACS/EEG) neuroplasticity assessment. EMG: electromyography; MEP: motor evoked potential; TBS: theta-burst stimulation.

An index of the duration of the theta-burst stimulation (TBS)-induced modulation of corticospinal excitability was defined for each participant. Participants were set up in a chair with electromyography (EMG) electrodes placed on the first dorsal interosseus on the dominant hand to measure motor threshold in the corresponding (contralateral) motor cortex (Figure 5A). The motor threshold serves as the basis for the following TMS measures over the motor cortex. When administering TMS, the figure of 8 coil was placed tangentially to the scalp with the handle pointing posterior for all stimulations. We proceeded with determining motor threshold and conducted TMS (single pulse TMS and TBS) over the dominant motor cortex. After removing the EMG equipment from the participant, a post-TMS safety evaluation was conducted. During each of the TMS/plasticity visits, the following procedures took place:

- Single pulse TMS was delivered using a biphasic figure of 8 coil. This stimulation phase consisted of 3 batches of 30 TMS pulses delivered every 5 to 7 seconds at 120% of resting motor threshold prior to TBS stimulation as a baseline. Resting motor threshold was defined as the minimum stimulus intensity that produced a small motor evoked potential (MEP; about 50 μV in 50% of 10 trials) during relaxation of the tested muscles.
- For this study, participants received iTBS over the motor cortex. iTBS consists of bursts of 3 pulses at 50 Hz repeated at intervals of 200 ms for a total of 2 seconds (1 train); each train were repeated every 10 seconds for 20 times for a total of 600 stimuli. After TBS is applied to the motor cortex in an intermittent fashion (iTBS), TMS-induced potentials show increased amplitude for a period of 20 to 30 minutes. Stimulation intensity was delivered at 80% of active motor threshold, defined as the minimum stimulus intensity that produces a small MEP (about 200 μV in 50% of 10 trials) during isometric contraction of the tested muscles, at about 20% of maximum voluntary contraction. After iTBS has been administered, batches of single pulse TMS were administered at timed intervals for 30 minutes over the motor cortex (T5, T10, T20, and T30). The degree of potentiation of MEPs (ie, the percentage change in peak-to-peak MEP amplitude from baseline at each post-iTBS time point (T5, T10, T20, and T30) was considered as a TMS index of neuroplasticity [21-23].

##### tACS/EEG Plasticity

All visits were monitored remotely using a secure, video platform through Zoom and conducted by the study team members of the laboratory, with appropriate oversight. In this paradigm, small electrodes placed on the scalp deliver a short burst of tACS, which induces a controlled perturbation in the brain that is measured by EEG changes (Figure 5B). tACS/EEG is uniquely suited for a home-based intervention as it can be delivered with a device designed for home use. Using established procedures via a secure video platform, the participant was instructed on how to appropriately place the EEG cap, and the study investigators conducted the assessment via remote neurophysiological monitoring. An index of change in the tACS-induced modulation of cortical excitability was defined for each participant, and brain functional connectivity and interregional coordination were directly estimated from EEG. We used established tACS/EEG procedures to measure neuroplasticity [16,17]. We used the noninvasive brain stimulation/EEG hybrid Starstim-Home system (Neuroelectrics) and recorded EEG before (5 minutes), during (3 minutes), and after (5 minutes) tACS stimulation. Participants received tACS stimulation (3 minutes, 40-60 Hz, 1-2 mA) targeting the motor and prefrontal cortices through remote neurophysiologic monitoring by the study team. We used published methods of artifact removal and event detection algorithms to extract the magnitude and duration of post-tACS gamma EEG power changes from the EEG signals [16,17]. In accordance with the safe application of neurophysiological measures recommended by the International Federation of Clinical Neurophysiology, we collected and monitored adverse events.

#### Cognitive Function

##### Visuomotor Processing Speed and Cognitive Flexibility and Task Switching

This was assessed using the Trail Making Test Part B [24,25]. Trail Making Test Part B consists of encircled numbers (1-13) and letters (A-L). Individuals were asked to connect the circles alternating between numbers and letters as quickly as they could without making mistakes. The test was scored according to the number of errors made and the time (in seconds) to completion.

##### Response Inhibition, Mental Flexibility, and Attentional Control

This was assessed using the Stroop Color and Word Test [26]. The test consists of 3 components, each component consisting of 100 items, presented in 5 columns. In the first portion, participants were asked to read words printed in black ink (RED/GREEN/BLUE). In the second portion, participants were asked to identify the color of ink in which a series of Xs are printed. In the final section, participants were asked to identify the color of ink the color words were printed in. Participants were asked to complete each component as quickly and accurately as possible. Each section was scored based on number of items completed accurately within a 45-second period.

For the remote group, the same cognitive battery minus the Stroop color and word test and with the addition of a TestMyBrain.org [27] battery of Digit Symbol Matching, Simple and Choice Reaction Time, and Gradual Onset Continuous Performance Test was used via a secured Zoom for Healthcare platform.

##### Attention and Working Memory

The Digit Span subtest of the Wechsler Adult Intelligence Scale, 4th Edition [28] was used to asses this construct. This measure consists of 3 parts: Digit Span Forward, Digit Span Backward, and Digit Span Sequencing. Participants were read increasingly longer lists of numbers (starting with 2 digits and increasing in difficulty up to 9 digits) and asked to repeat them verbatim, in backward order, and sequentially (ie, from smallest to largest) on Digit Span Forward, Digit Span Backward, and Digit Span Sequencing, respectively. Each portion was discontinued when participants could not accurately repeat 2 consecutive trials of equal difficulty.

##### Global Cognition

The Repeatable Battery for the Assessment of Neuropsychological Status Update [29] is a brief battery used to measure an individual’s cognitive state. The assessment was completed in approximately 25 minutes. The battery was composed of 5 cognitive domains:

- Immediate memory was assessed through a list learning task of 10 words and a short story. Participants were asked to repeat a list of 10 words over a series of 4 learning trials. The score was dependent on the number of correctly recalled words over the 4 trials. Participants were then read a short story and asked to recall as many details as possible from the story that they could remember over 2 trials. The score was based on the number of correct key details the participant could recall.
- Visuospatial was assessed using a figure copy and line orientation task. Participants were asked to copy a figure as accurately as they could while being timed. The score was based solely on the exactness of the copy of the figure. Participants were then shown 13 lines that were numbered 1 to 13 and arranged in a fan shape that were 15 degrees apart from each other at the top of a page. Participants were shown a series of 2 lines, isolated from the fan shape, and asked to identify what numbers they corresponded to on the fan shape. Participants were awarded 1 point for each time they correctly match the lines.
- Language was assessed through picture naming and semantic fluency. Participants were asked to identify the name of 10 different figures. The score was based on the number of figures the participant named correctly. Participants were then asked to name as many items from a specific category as they could for 1 minute. One point was awarded for each unique response.
- Attention was composed of a digit span and coding task. Participants were read a series of numbers increasing in length that they were asked to repeat. Participants were awarded 1 point for each series of number they correctly recalled. Coding was comprised of a key of 9 symbols that were associated with a number 1 through 9. The participant was then asked to use the key to assign numbers to a list of matching symbols below, where the numbers were missing. One point was given for each symbol correctly assigned to a number based on the key.
- Delayed memory was measured by recalling the 10 words and short story from the immediate memory task, recalling the figure copied during the visual spatial constructional task, and identifying the 10 words from the list learning task among 20 other words.

##### Verbal Fluency

The Verbal Fluency subtest of the Delis-Kaplan Executive Function System [30] was used to assess verbal fluency. This subtest comprised letter fluency, category fluency, and category switching. During the letter fluency portion, participants were asked to name as many words as they could that began with a certain letter of the alphabet within 1 minute. There were 3 trials with 3 different letters, and the participant was awarded 1 point for each unique, appropriate response. For category fluency, participants were asked to name as many items as they could that corresponded to the category provided within 1 minute. Participants completed 2 trials with 2 different categories and were awarded 1 point for each unique, appropriate response. During the category switching, the participant was asked to switch between naming items from 2 different categories within 1 minute. Participants were awarded 1 point for each unique, appropriate response given for each category as well as each time they correctly switched between categories.

##### TestMyBrain.org

As there were some tests that were not appropriately delivered via Zoom, a few additional tests were used for option B. A TestMyBrain link was sent to participants during their assessment visit, and the investigator guided them through the test. Overall, this portion of the test required 10 minutes. The test comprised the following:

- Processing speed was assessed using the TestMyBrain Digit Symbol Matching. Participants were shown symbols that matched a corresponding number. Participants quickly and accurately selected the appropriate number. They had 90 seconds to complete as many symbols as possible.
- Basic psychomotor response speed was measured with TestMyBrain Simple Reaction Time. Participants were shown either GO! or WAIT! Participants pressed the space bar on the computer as rapidly as possible only when the word GO! was presented.
- Choice Reaction Time was used to measure processing speed, response inhibition, and attention. Participants were shown 3 boxes with arrows inside of them. Two boxes had the same color, and one was different (odd color). Participants must rapidly select the direction of the arrow in the odd colored box.
- Sustained attention, response inhibition, and cognitive control was measured with the Gradual Onset Continuous Performance Test. Participants were asked to press a key when a city picture appeared and to not press when a mountain image appeared. The images rapidly transition from one to the next with mountains appearing only 10% to 20% of the time.

#### Cardiorespiratory Fitness

##### Incremental Shuttle Walking Test

**Figure 6:**
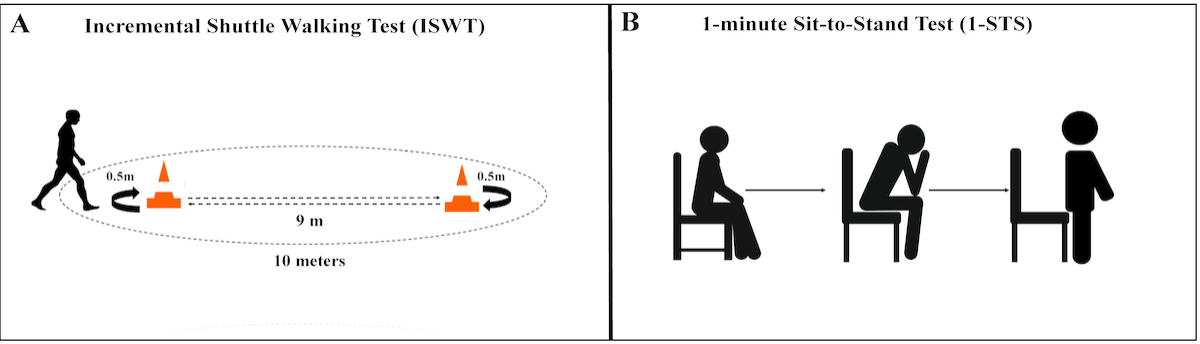
Cardiorespiratory fitness assessment.

The Incremental Shuttle Walking Test was performed to determine cardiorespiratory fitness by evaluating maximal walking velocity and walking distance during the test (Figure 6A) [31]. These values were used to estimate peak oxygen consumption (VO_2_ peak) changes over time as a measure of aerobic capacity [32]. Prior to the test, participants were fitted a heart rate monitor (Polar H10, Polar Electro Inc), seated in a comfortable chair for 5 minutes, and we measured blood pressure, heart rate, rate of perceived effort, and oxygen saturation. Participants were screened for signs and symptoms (eg, dizziness, nausea, chest pain) and a list of medications taken before the test. Participants were then instructed to walk progressively a distance of a 10-meter course around a marking between 2 cones, keeping to the speed indicated by the bleeps on the audio recording. At every minute, the participants had to increase their speed to keep up with the test. We recorded reasons for termination, and these included the following: (1) participant became too breathless to maintain the required speed or could no longer keep up with the set pace, (2) operator determined that the participant was not fit to continue (eg, reached 85% of predicted maximum heart rate), or (3) operator assessed that the patient was unable to sustain the speed and cover the distance to the cone prior to the beep sounding. Participants were closely monitored upon completion and 5 minutes after test cessation, including blood pressure measurement, heart rate, oxygen saturation, and rate of perceived effort. Heart rate recovery was also recorded at minute 1 and minute 2 after test cessation.

##### 1-Minute Sit-to-Stand Test

We adapted our exercise assessment to a submaximal aerobic capacity assessment using the 1-Minute Sit-to-Stand Test (Figure 6B) [33], which could be completed in the participant’s home and monitored securely through the Zoom platform. The 1-Minute Sit-to-Stand Test was performed to determine maximal repetitions completed in 1 minute. Participants were asked to join a Zoom meeting where they were asked to confirm their current geographical location and emergency phone number. Prior to the start of the test, the investigator requested participant place a sturdy chair in the camera view range in a safe location for proper monitoring, preferably an armless chair of standard height (45.0 to 48.0 cm) placed against a wall for stability. The participant was asked to don the Polar Heart Rate monitor and an activity monitor (ActiGraph GT9X Link) and have a seat in the chair. After this, vitals were taken after 3 to 5 minutes of seated rest, and the investigator completed a signs and symptoms check. Blood pressure, heart rate, perceived exertion, and oxygen saturation were documented by the investigator. The participant was then asked to stand up and sit down from a sturdy chair for 1 minute while being monitored by a trained study member. The number of sit-to-stands performed in 1 minute was recorded. The participant’s heart rate was monitored continuously throughout the test, and heart rate and blood pressure was taken and documented immediately after the assessment and after 5 minutes of seated rest and was continued monitored until the participant returned within 20% of their baseline measurement. Heart rate recovery was also recorded at minute 1 and minute 2 after test cessation.

In the remote group, we were also able to measure heart rate variability. Heart rate variability was assessed at rest and during the 1-minute Sit-to-Stand Test using the ActiGraph GT9X Link, paired with a wireless heart rate monitor (POLAR Inc) that generated beat to beat intervals (RR interval), an approach demonstrated to be comparable to an electrocardiogram for heart rate variability measurement [34]. For analysis, data will be then transferred to a computer using the CentrePoint system. All RR data will be manually reviewed by trained team members. Heart rate variability measures will include time and frequency domains and spectral and nonlinear indices [35,36], calculated offline using Kubios and Cardioedit/Cardiobatch software [37].

### Secondary Outcome Measures

#### Functional Mobility and Postural Control

Participants underwent a functional evaluation to assess the influence of physical exercise on gait and mobility, postural control, and cognitive-motor interference.

#### Functional Assessment (Gait and Mobility)

Participants were outfitted with wireless accelerometers (Mobility Lab System, APDM Wearable Technologies Inc) secured with Velcro straps to the wrists, ankles, sternum, and lower back. Then, the Timed Up-and-Go (TUG) test, 90-second trials of walking, and standing postural control were administered following these procedures:

- The TUG test is a valid and reliable test of mobility [38] that requires participants to stand from a chair on command, walk 3 meters, turn around, walk back to the chair, and sit back down. The TUG was performed under 3 conditions: 2 trials of normal walking (regular pace) TUG single-task, 2 trials of fast walking TUG single task, and 2 trials of TUG dual task. For the TUG dual task, the participants were given a 3-digit number at the beginning of the trial and were instructed to perform serial 7 subtractions while walking during the test. For the TUG dual task, a dual-task effect was computed as the percentage change of the TUG dual task from the TUG single task, and the cost of the cognitive task in combination of the motor task was used to reflect a cognitive-motor interference [39].
- Four trials of the 90-second walking were completed along a 35×4 m indoor hallway to assess gait under normal and cognitive dual-task conditions. Cognitive dual-task conditions included dual-task walk, dual-task walk with priority on walking, and dual task with priority on the cognitive task. The cognitive dual task was to verbalize serial subtractions of 7 from a random 3-digit number.
- Postural control was assessed by measuring postural sway (ie, center-of-pressure fluctuations) during six 30-second trials of standing—2 with eyes open, 2 with eyes closed, and 2 while performing the serial 7 subtraction cognitive task.

#### Remote Group

For the remote option, we performed the TUG test as a measure of functional mobility in all 3 conditions described above: normal TUG single task, fast TUG single task, and TUG dual task. The test was completed and supervised via a secured Zoom platform. The participant was instructed to set up the chair and measure a 3-meter space with the provided tape measure and masking tape to complete the assessment in their home. The investigator guided the TUG procedures and recorded their performance.

#### Blood Collection and Genetic Testing

Blood samples were collected to assess peripheral BDNF levels (pg/mL), VEGF (pg/mL), and high-sensitivity C-reactive protein (mg/L) concentrations. We also evaluated DNA to assess BDNF, Val66Met polymorphism, and the presence of apolipoprotein E e4 allele. We stored blood samples for future examination of other potential genetic, epigenetic, metabolic, or proinflammatory markers. Blood samples were delivered to the lab on ice within 2 hours of collection, centrifuged, and stored at –80 °C until processing. The blood collection was only performed on the in-person participants.

#### Physical Activity and Exercise

The following physical activity and exercise questionnaires were applied for both in-person and remote groups.

#### Lifetime Physical Activity Questionnaire

The Lifetime Physical Activity Questionnaire [40] uses questions about participant physical activity at various times in the participants’ life. Participants were asked to estimate the average amount of time each week and the average number of months each year spent in moderate, vigorous, and other activities.

#### Exercise Self-Efficacy

The Exercise Self-efficacy Questionnaire [41] assessed how sure participants were that they would perform exercise under different conditions or constraints (eg, how sure you are that you will exercise when you are feeling tired). Each item responses varied from 1 (not at all sure) to 4 (very sure).

#### Exercise Barriers and Motivators

Exercise Barriers Questionnaire [42] assessed participant agreement of a list of statements about barriers to physical exercise (eg, I am not sufficiently physically active because I haven’t any time for physical activity.). Each item responses varied from 1 (disagree), 2 (partially agree), and 3 (agree). Participants were also asked to give reasons that help them remain sufficiently physically active (eg, I have exercise equipment at home).

#### Remote Group

Questionnaires were completed by the participant using a REDCap link sent via a secured email. We also objectively collected physical activity data with the ActiGraph GT9X-BT Link.

#### Physical Activity

An objective measurement of overall physical activity was collected using the activity monitor ActiGraph GT9X-BT Link, the gold standard, research-grade, activity monitor. The device includes a 3-axis gyroscope, magnetometer, and accelerometer and provided raw data on a variety of objective physical activity, wear compliance, and sleep measures using validated algorithms through the CentrePoint platform. The physical activity raw data included (1) daily activity profile (steps taken, kcals, and activity counts), (2) activity and sedentary bouts (bouts of sustained physical activity), and (3) activity intensity (the time spent within different categories of activity intensity (sedentary, light, moderate-to-vigorous). The CentrePoint is a cloud-based data capture and management platform technology that permits participants to transfer data to the cloud via computer or mobile, and investigators had the ability to monitor patients remotely and in near real time to assure they were engaged and compliant with the protocol. The device was worn on the waist. We collected data at baseline during 3 to 5 days before exercise intervention and daily activity data during the intervention.

#### Wear Compliance

The participants were advised to use the activity monitor all day long, including sleep time, and only remove it when taking a shower or swimming. Wear compliance was monitored with the daily amount and percentage of time the participant wore the activity monitor. The data were validated if the participant wore the monitor for a minimum of 12 hours.

#### Sleep

The Pittsburgh Sleep Quality Index is a valid and reliable measure broadly used in clinical and research setting that evaluates sleep quality over the last month, providing an index of severity and nature of the sleep disorder [43,44].The Pittsburgh Sleep Quality Index was assessed in both in-person and remote protocols.

In the remote group, sleep quality was assessed using the ActiGraph GT9X-BT Link. Daily raw data were collected at baseline and during the intervention. Sleep data included total sleep time, wake after sleep onset, and sleep efficiency.

#### Brain Health Index

We expanded the questionnaires to include a structured survey used to assess the 7 pillars of brain health (cognitive function, physical exercise, nutrition, comprehensive health, socialization, sleep, and vital plan [perceived meaning in life]) developed by the Barcelona Brain Health Initiative [45]. The questionnaire includes 52 items with yes or no responses. A percentage weighted score was used to indicate the brain health index of the participants.

#### Depression

For both options (remote and in-person), we used the full version of the Geriatric Depression Scale. The Geriatric Depression Scale is a valid and reliable self-reported measure of depression in older adults and contains a total of 30 items [46].

#### Study Feasibility and Adaptability

##### Adherence to the Intervention

The adherence rate was calculated in both groups by the (1) proportion of participants who initiated the intervention out of the total allocated, (2) proportion of participants who completed the whole planned intervention out of the participants who initiated the intervention, and (3) total number of days to complete the intervention from exercise session 1 to session 24. In both groups, we also assessed the proportion of participants lost to follow-up, proportion of participants who withdrew, and proportion of participants who rescheduled sessions and the average number of rescheduled sessions.

##### Exercise Intensity Adherence

We calculated the proportion of time spent within the planned target heart zone (moderate and vigorous intensity) for each session (24 in total), for each participant, and the average of participants for each group.

##### Adaptability of the Intervention and Assessment Methods

This was assessed by comparing the outcome results of both groups, and the feedback of the participants. At the end of the study, participants completed an exit interview that included questions on their feedback about the intervention and methods. Examples of specific questions: How difficult was it for you to use Zoom for testing and exercise sessions? Did you have any difficulties with the remote program, connectivity, or any of the technology used within the study?

##### Technology Compliance

This was assessed in the remote group by evaluating the compliance report of the activity monitor ActiGraph GT9X Link generated by the CentrePoint platform: (1) daily use at baseline and during the intervention, (2) daily validated data (minimum of 12 hours), and (3) average of daily hours.

#### Participant Self-Evaluation, Study Feedback, and Plans for Exercise

After study completion, participants completed an exit interview containing questions in the following domains: (1) motivation to join the study, (2) likelihood they would continue exercise, (3) their plan to continue exercising, (4) types of exercise they planned to do, (5) confidence to exercise on their own, (6) dietary changes during the study, and (7) changes experienced during the course of the intervention (eg, energy level, thinking, memory, motivation, fitness, and others).

We also asked their feedback about the study. Questions related to (1) health problems experienced, (2) importance of procedures adopted by the study team (eg, trainer, interactions during session, flexibility of schedule), and (3) what they liked about the study (eg, being a part of a research study, making a commitment to exercise).

For the remote group, we asked about their experience with this modality of study, including (1) difficulty with the remote procedures (eg, using the computer and other technologies) and (2) recommendations and suggestions.

### Sample Size

With a significance level of .05, an estimated sample of 80 individuals would provide 80% power to detect a Cohen effect size of 0.36 using a 2-sided paired *t* test for aim 1, the effect size associated with improvements in executive function in a recent meta-analysis of healthy adults aged older than 55 years [47], and an attrition of 20%, not uncommon in exercise intervention trials. In addition, a sample of 80 participants would provide 80% power to detect a correlation of 0.34 between the change in TMS neuroplasticity and the change in cognitive performance for aims 2 and 3 and 80% power to detect an *R*^2^ of .11 attributed to a genetic modification in the change in cognitive performance using an *F* test for aim 4.

### Statistical Analysis

Baseline characteristics will be summarized as means and standard deviations or medians and interquartile ranges for continuous variables and as frequency and percentage for categorical variables. Normality for the distributions of continuous variables will be visually assessed and statistically tested. If the normality assumption is questionable, Box-Cox transformation, a logarithmic or square root transformation, will be used to reduce the skewness in the indicated analyses or nonparametric tests such as Wilcoxon rank test will be used. All statistical tests will be conducted against a 2-sided alternative hypothesis with a significance level of .05.

In the univariate analysis, we will compare the cognitive performance (overall and by domain) before and after exercise using paired *t* test to test for specific aim 1. For specific aim 2, we will compare neuroplasticity response and cardiorespiratory response before and after exercise using a mixed effects repeated measures model. To account for potentially important covariates in the model (eg, age, sex, race/ethnicity, education), supporting analyses will control for these factors in a non–time-varying fashion. For specific aims 3 and 4, we will compare mean change in cognitive performance across subgroups by genotype using an *F* test. In the multivariable analyses, we will adjust for baseline characteristics (age, sex, race/ethnicity, education) using mixed effect modeling to quantify improvements in cognitive performance after exercise (specific aim 1) and determine an association between changes in neuroplasticity, cognitive performance, and changes in cardiorespiratory response (specific aim 3). We will use modification models by including interaction terms (time-by-genotype) to explore the relationship between change in cognitive performance after exercise and BDNF levels, Val66Met, and APOE e4 status (specific aim 4).

All data entry will be coded and double-entered into an Excel (Microsoft Corp) spreadsheet for analysis. Exploratory analyses, plotting, and mixed effects regression analyses will be performed using Stata statistical software (version 17 or later, StataCorp LLC).

## Results

This study was funded by award number KL2TR002737 from the National Center For Advancing Translational Sciences of the National Institutes of Health in September 2018 for 2 years, and the protocol was first approved by the University of Miami IRB in November 2018. The modification of the study adaptation for a remote version was approved by the IRB in July 2020. Participants were recruited using an open enrollment process. A total of 254 individuals were screened for eligibility, and 75 adults aged 55 years or older were recruited from August 2019 to April 2021. A total of 41 individuals were enrolled in the in-person protocol, and data were collected between February 2019 and March 2020. A total of 34 individuals were enrolled in the remote study, and data were collected between August 2020 and April 2021. Currently, data are being processed and analyzed. Results are expected to be published in 3 major papers in 2021 and 2022.

## Discussion

The research proposed in this investigation will advance the field of exercise and cognition by generating evidence on the mechanistic action of 8 weeks of moderate to high exercise on cognitive performance, neuroplasticity and cardiorespiratory function, and examining potential effect modification of cognitive response to exercise by endothelial function and genetic factors. The data generated in these studies will introduce tangible parameters to guide the development of personalized exercise prescription models for maximal cognitive benefit in aging adults. Successful completion of the specific aims will enable researchers to acquire the appropriate expertise to design and conduct studies testing personalized exercise interventions in person and remote delivered, likely to be more effective at promoting cognitive health in aging adults.

Neuroplasticity assessments are critical to understanding why some people show greater cognitive response to exercise than others do, which will then facilitate development of optimal exercise doses and regimens to achieve maximal cognitive benefits for each person. Exercise is a low-cost intervention with an established safety profile, so in addition to advancing scientific knowledge, the results of the proposed study have the potential for direct translation into community settings and eventually to health care policy.

With the addition of the remote group to the study, we intended to gain valuable data in exercise adherence and feasibility of the introduction of effective technology aimed at improving cognitive brain health in the aging population. Given the increasing use of ubiquitous mobile technologies in aging and older adults, especially considering the post–COVID-19 pandemic effects, the addition of a remotely delivered clinical research program facilitated recruitment, addressed barriers to participation, and accelerated the translation of findings to a broader audience and clinical application.

## Appendix

Multimedia Appendix 1 Study booklet sample.

Multimedia Appendix 2 Peer-review report from the Miami CTSI KL2 Award Committee.

## Abbreviations

BDNF: brain-derived neurotrophic factor
EEG: electroencephalography
EMG: electromyography
HR: heart rate
IRB: institutional review board
iTBS: intermittent theta-burst stimulation
MEP: motor evoked potential
REDCap: Research Electronic Data Capture
tACS: transcranial alternating current stimulation
TBS: theta-burst stimulation
TMS: transcranial magnetic stimulation
TUG: Timed Up-and-Go
VEGF: vascular endothelial growth factor
