# Supplementary material for: Harnessing Neuroplasticity to Promote Brain Health in Aging Adults: Protocol for the MOVE-Cog Intervention Study
Source: JMIR Res Protoc. 2021 Nov 23;10(11):e33589. doi: 10.2196/33589 (PMC8663452; doi:10.2196/33589)
Supplement: Multimedia Appendix 1 [file resprot_v10i11e33589_app1.pdf]

UNIVERSITY OF MIAMI

Exercise for brain health

Welcome to our exercise study! This is your participant's booklet that will help you go through the study

To start, it is important to keep in mind that a healthy lifestyle is made up of many different components: physical, emotional, mental, and social health. In this context, there is a wealth of evidence supporting the role of physical exercise as a powerful health-promoting agent.

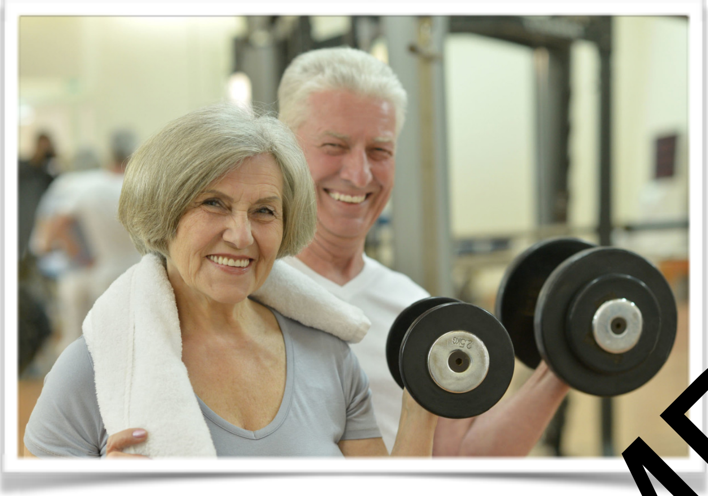

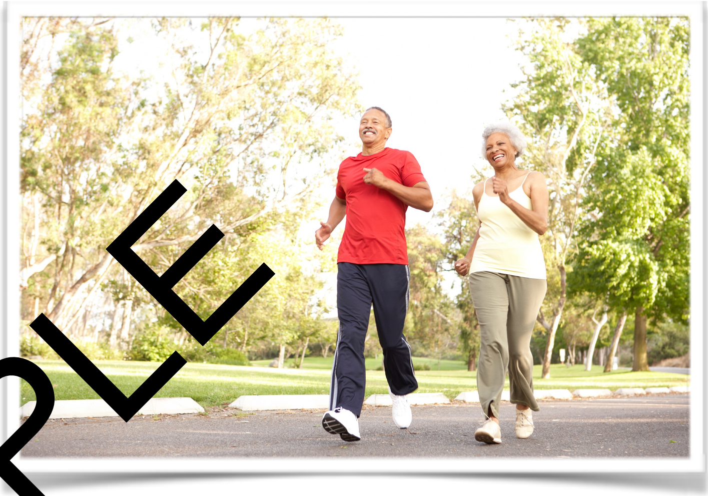

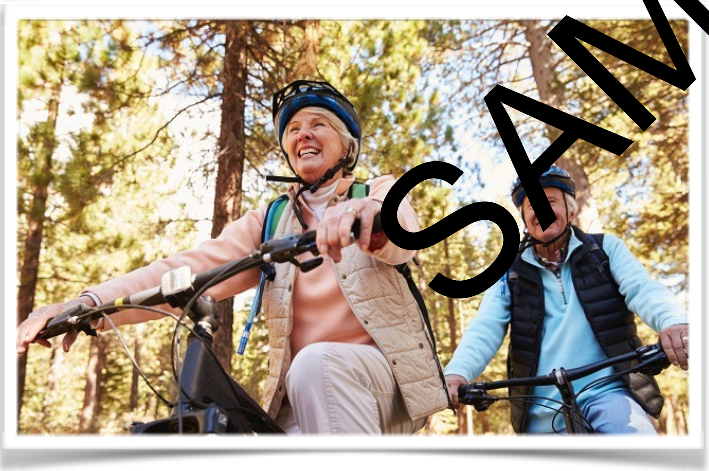

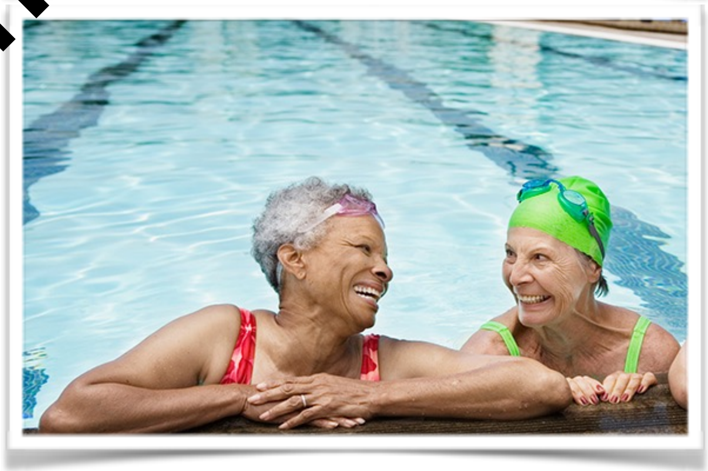

SAMPLE

The importance of being physically active

Warming up the brain…

We know that regular exercise contributes to a healthy lifestyle by decreasing the risk of heart disease and strokes, high-blood pressure and diabetes, and improving mood. In addition, several studies suggest that regular exercise is also very important for keeping the brain healthy during the many changes that occur throughout our lifetime. As we grow older, there are physiologic (natural) changes in the structure and function of the brain that result in changes in cognition.

Participant

NAME:

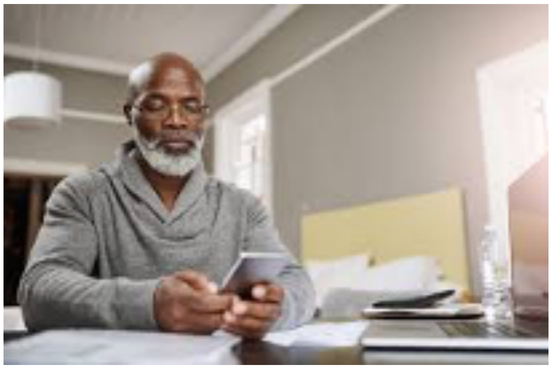

Neuromotor Plasticity Lab Contact Information

Call or Text us at : (305) 912-7871

Track your reading...

SAMPLE

IN THIS BOOKLET, YOU WILL FIND INFORMATION AND SPECIFIC RECOMMENDATIONS ON HOW TO FOLLOW ALONG THE STUDY.

- Section 1: Refreshing the study Purpose — Page 3
  - Defining important terms — Page 3
- Section 2: Study Kit — Page 4
  - Section 2.1 Blood pressure monitor — Page 5
  - Section 2.2 Heart rate monitor — Page 6-7
  - Section 2.3 Pulse oximeter — Page 8
  - Section 2.4 Physical activity monitor — Page 9-11
  - Section 2.5 Tape measure, masking tape, alcohol prep pads — Page 12
  - Section 2.7 Kit safe care and return procedures — Page 13
- Section 3: Getting Started — Page 14
- Section 4: Pre Testing — Page 15
  - Section 4.1 Fitness and Walking Test — Page 15
  - Section 4.2 Cognitive Testing — Page 16
- Section 5: Daily Exercise Sheet — Page 17-40
- Section 6: Post Testing — Page 41
  - Section 6.1 Fitness and Walking Test — Page 41
  - Section 6.2 Cognitive Testing — Page 42

Section 1. Study Purpose

THE OVERALL GOAL OF THE PROPOSED STUDY IS TO EVALUATE THE EFFECTS OF AN 8-WEEK AEROBIC EXERCISE PROGRAM ON COGNITION AND DETERMINE THE RELATIONSHIP BETWEEN FITNESS, COGNITIVE IMPROVEMENTS AND NEUROPLASTICITY.

Let's define some important terms

COGNITIVE AGING

"It is not a disease or a quantifiable level of function. It is a process of gradual, ongoing, yet highly variable changes in cognitive functions that occur as people get older."

Institute of Medicine Report on Aging, 2015

COGNITION

"Refers to the mental functions involved in attention, thinking, understanding, learning, remembering, solving problems and making decisions."

Institute of Medicine Report on Aging, 2015

SAMPLE

COGNITIVE IMPAIRMENT

"An intermediate stage between cognitive aging and probable initial diagnostic of dementia. Individuals may present with a variety of cognitive symptoms, but these do not limit their ability to perform daily life tasks"

Petersen et al, 1999

PHYSICAL EXERCISE

"The execution of activities in a planned, structured manner to maintain or increase physical fitness, health and wellness."

Williams et al, 2010

PHYSICAL FITNESS

"It is a state of health and well-being, and refers to the ability of your body systems to work together efficiently to allow you to be healthy and perform activities of daily living. Being efficient means doing daily activities with the least effort possible."

NEUROPLASTICITY

"Also known as brain plasticity, is the ability of the brain to undergo biological changes (flexibility) and allows our brain to undergo great changes in response to our experiences, and as a result, our brain is constantly adapting and learning"

Section 2. Study Kit

You are receiving a STUDY KIT including items that have been previously sanitized using alcohol-based solution greater than 70%. The study kit includes:

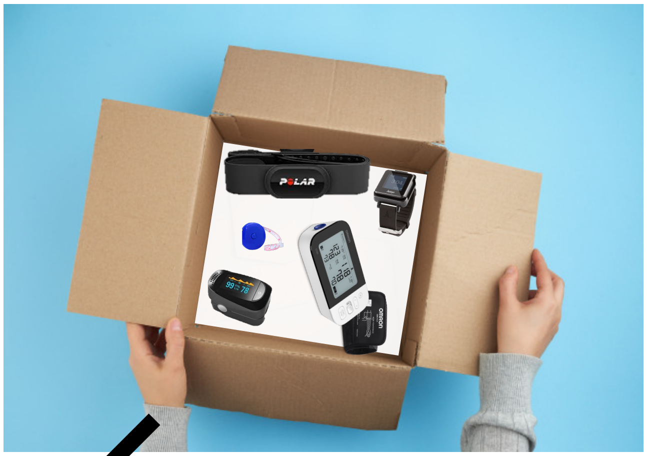

1. **a blood pressure monitor** (OMRON BP7350)
2. **a heart rate monitor** (Polar H10)
3. **a pulse oximeter** (Diagnostix™ 2100 Fingertip Pulse Oximeter)
4. **a physical activity monitor** (Actigraph GT9X-BT Link)
5. **a tape measure, masking tape, alcohol prep pads** Isopropyl Alcohol 70%
6. a **pre-paid return shipping label**

When the study kit arrives, before beginning any assessments or intervention, we will schedule a remote familiarization session meeting with all of the tools in the study kit. You will learn to take your own blood pressure, blood oxygen levels, put on the heart rate monitor, and proper safety and cleaning procedures. If needed, we may schedule multiple meetings to ensure you are properly familiarized with all of the materials in the study kit.

SAMPLE

Section 2.1 Blood pressure monitor

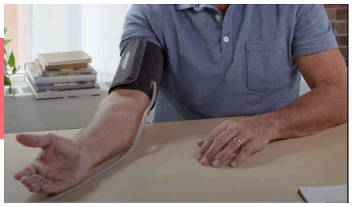

WHAT IS BLOOD PRESSURE?

In the most basic sense, blood pressure is a way of measuring how much force is being exerted on the walls of your blood vessels (artery) as blood flows through them.

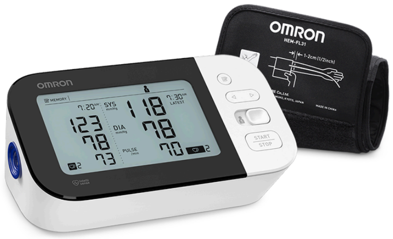

1. **Systolic** is the top number. It represents the pressure as your heart contracts to pump blood to the body

2. **Diastolic** is the bottom number. It represents the pressure between beats, when your heart relaxes.

WHAT IS A DIGITAL BLOOD PRESSURE MONITOR?

The digital blood pressure monitor is an accurate, easy to use and reliable equipment that measures your blood pressure before, during, and after exercise.

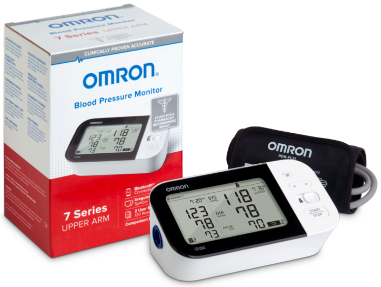

Products included in the box: monitor, easy wrap cuff, 4 "AA" batteries, instruction manual, a quick start guide, and an AD adapter. See manual for more information or contact us if you have any question.

HOW DO I MEASURE MY BLOOD PRESSURE?

SAMPLE

1. Plug the arm cuff into the monitor securely until it clicks. **(Figure 1)**
2. Remove tight fitting clothing from your upper arm (left arm preferred).
3. Sit in a comfortable chair with your back and arm supported and feet flat on the floor (legs uncrossed). Rest your left arm on a table so that the cuff is at the same level as your heart. Turn the palm of your hand upward. **(Figure 2)**
4. Put the cuff on your upper arm.
5. Align the marker on the cuff over the brachial artery on the inside of your arm. **(Figure 3)**
6. The cuff tube should run down the center of the arm even with the middle finger.
7. The bottom of the cuff should be approximately ½-1 inch above the elbow.
8. Secure the cuff around your arm making sure i is firmly in place.
9. Relax your arm and press start on the machine.

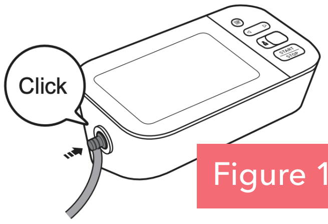

Figure 1

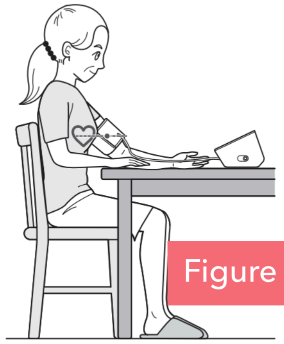
Figure 2

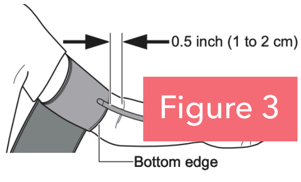

Figure 3

SECTION 2.2 Heart rate monitor

WHAT IS HEART RATE?

Heart rate, also known as **pulse**, is the number of times a person's **heart beats** per minute. Measuring your heart rate is any easy way to gauge your health, as it provides a real-time snapshot of your heart muscle function.

WHAT IS A HEART RATE MONITOR?

It is a device worn in proximity to the heart that gathers electrical activity data to derive your heart beats per minute. The heart rate monitor is used to capture and transmit real time heart rate information during the study.

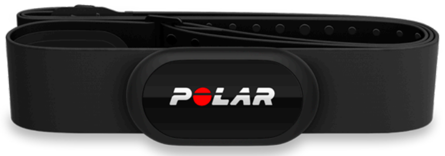

Products included in the box: Polar H10 heart rate sensor, Polar Pro chest strap, Polar H10 getting started guide

HOW DO I MEASURE MY HEART RATE?

First Step: Putting on the heart rate monitor

1. Thoroughly wet the electrode areas on the strap with water. It is important that the electrode area is sufficiently moist so that a good skin-to electrode connection is made. **(Figure 1)**
2. Snap the pod onto the strap. **(Figure 2)**
3. With the logo on the pod facing up, wrap the strap around your back and secure the strap. Center the pod so that it is located at the bottom of your breastbone (sternum). Adjust the strap to create a snug, yet comfortable fit. **(Figure 3)**

Figure 1

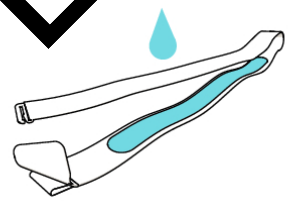

Figure 2

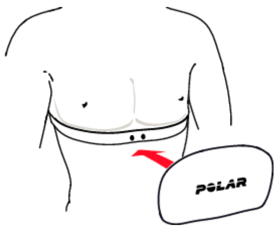

Figure 3

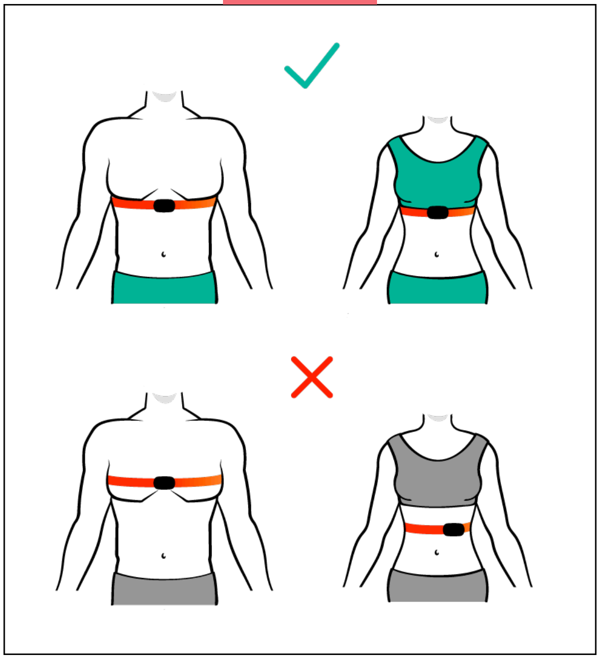

Second step: Pairing with the Actigraph

- Once in place, move within 5 to 10 feet of the Actigraph activity monitor device that has been configured to collect heart rate data. **You should be ready to go if using the activity monitor in your wrist.**

SAMPLE

SECTION 2.4 Physical Activity monitor

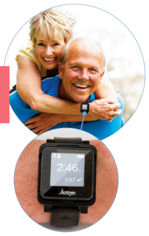

Figure 1

WHAT IS PHYSICAL ACTIVITY)?

World Health Organization defines physical activity as any bodily movement produced by skeletal muscles that requires energy expenditure – including activities undertaken while working, playing, carrying out household chores, traveling, and engaging in recreational pursuits. The term "physical activity" should not be confused with "exercise", which is a subcategory of physical activity that is planned, structured, repetitive, and aims to improve or maintain one or more components of physical fitness.

WHAT IS A PHYSICAL ACTIVITY MONITOR?

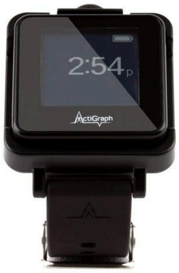

The activity monitor ActiGraph GT9X Link is the most sophisticated activity monitor available from the global leader in actigraph measurement. It can objectively measures physical activity, wear compliance, and sleep measures.

Products included in the box: ActiGraph monitor, link wrist band, single dock, USB cable, a getting started guide, a first band clip instruction guide.

HOW DO I CHARGE THE BATTERY?

Figure 1

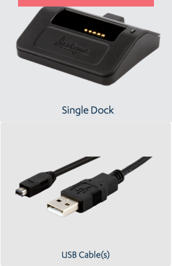

1.Connect the Link dock **(Figure 1)** to the computer [illegible] a wall outlet using the mini USB cable **(Figure [illegible])**.

2.Plug the ActiGraph Link into the do[illegible] with [illegible]the ActiGraph logo facing up **(Figure [illegible])**. O[illegible] connected, the LED light on the right side of th[illegible]ock [illegible]ill turn yellow **(Figure 4)**, indicating that is [illegible]rging.

3.Once the device is ful[illegible] charge[illegible] the yellow light will turn green and the batte[illegible] ic[illegible] the device will show as full and stay on steady **(Figu[illegible])**.

SAMPLE

Figure 2

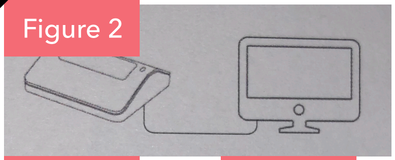

Figure 3

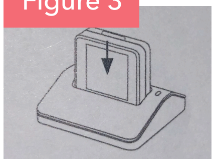

Figure 4

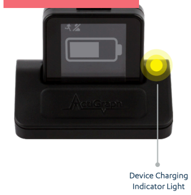

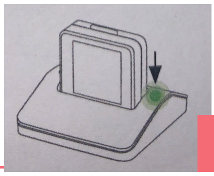

Figure 5

HOW DO I WEAR THE ACTIVITY MONITOR?

Figure 6

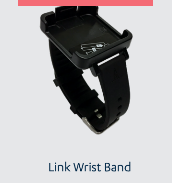

1.The ActiGraph Link should be worn at the **non-dominant wrist.** It is expected a daily wear compliance a minimum of 12 hours.

2.When the device is fully charged (steady green light), remove it from the dock and insert into the watch band as showing in the figure **(Figure 6)**.

2.1 Inserting the device: Position the plastic notch on the bottom edge of the device into the matching groove in the bottom, edge of the wrist band **(Figure 7)**. Gently push down on the upper part of the device until it snaps into place. **(Figure 8)**

2.2 Removing the device: Firmly grabs the deice with one hand while gently lifting the plastic tab on the top edge of the wrist band. **(Figure 9)** Lift up on the device until it pops out of the wrist strap. **(Figure 10)**

Figure 7

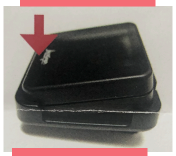

Figure 8

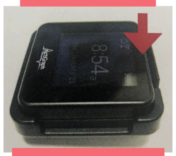

Figure 9

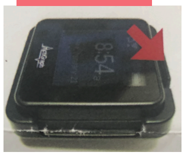

Figure 10

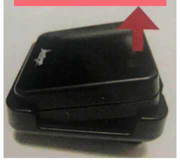

SECTION 2.5 Tape measure, masking tape, alcohol prep pads

TAPE MEASURE AND MASKING TAPE

The tape measure will be used only during the **Walking test.**

You will be asked to measure a 3-meter course (118.1 in or 9.8 feet) from the chair to a cone (bottle water). Use the masking tape to help with the marks. See following illustration.

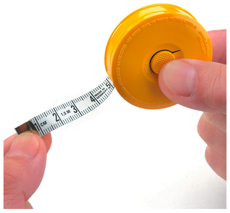

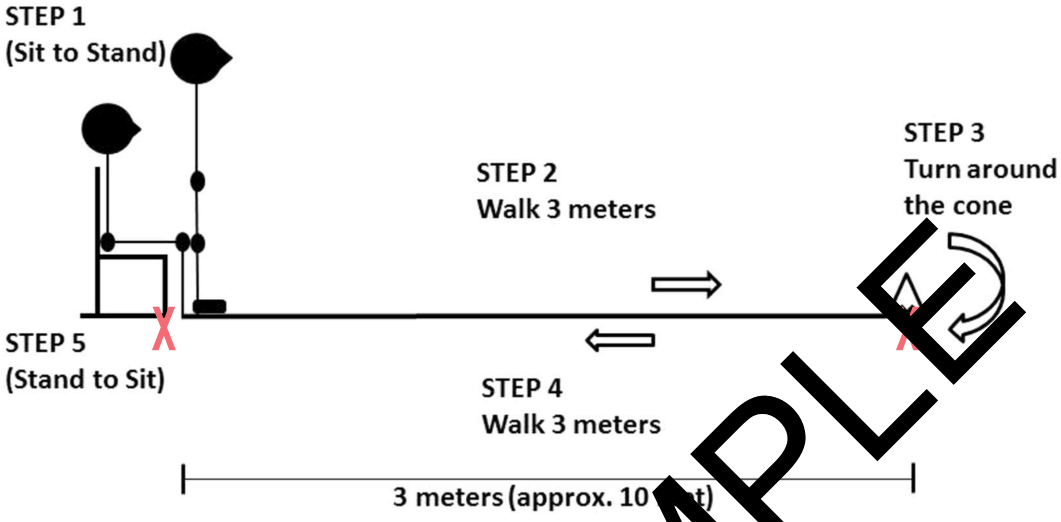

Place the masking tape in the floor as represented by the red X.

SAMPLE

ALCOHOL PREP PADS

All the equipment shipped to you will be previously cleaned properly.

You are required to clean the equipment after each usage as detailed herein and further explained by the study team.

1. Clean with alcohol the heart rate monitor after each usage (testing and exercise session). It is also advised to hand wash the straps using any commercial soap at least one time a week. After cleaning, place the straps in a ventilated area to dry.

2. Clean with alcohol the blood pressure monitor cuff after each usage.

3. Clean with alcohol the activity monitor and its band at least one time per week. Remember, the monitor must be removed during shower or swimming activities.

4. Clean inside surface of oximeter and your finger's tip with alcohol before and after use.

Important: Do not dry after cleaning. Let it dry by itself.

Section 4. Pre Testing

PRE TESTING

1. Fitness and Walking Test

2. Cognitive Testing

SECTION 4.1 Fitness and Walking test

Today we are going to do a fitness assessment. First, you will do an assessment where you must stand up from a chair, walk around an object, and return to sitting. Then you will do a fitness assessment, where you will stand-up and sit-down repeatedly for one minute.

Visit time: 1 hour

WHAT YOU WILL NEED:

1. [ ] Vitals Equipment: Blood Pressure Monitor, Pulse Oximeter

2. [ ] Polar Heart Rate Monitor

3. [ ] Actigraph

4. [ ] Sturdy Chair (open wall to place chair against or sturdy surface)

5. [ ] Tape Measure (10ft of space to walk)

6. [ ] Water Bottle or Cone

SAMPLE

Test Set-up

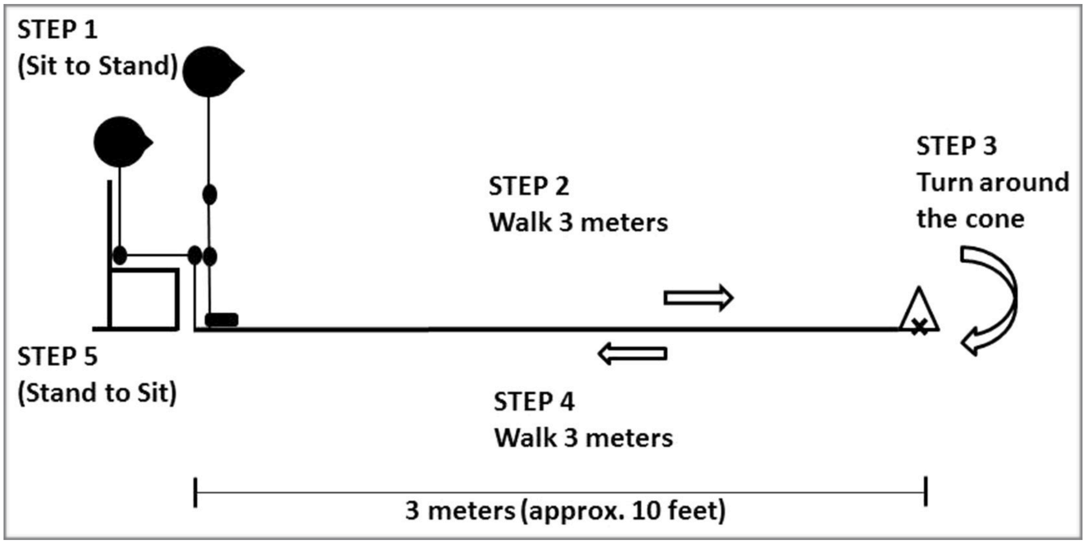

SECTION 4.2 Cognitive testing

You will be taking part in a cognitive test that will last about an hour and a half. During this test we will try to understand more about how your brain is working from a thinking perspective. We will test different areas of your cognition like memory. We will see if any of your thinking abilities change after the exercise intervention.

Visit time: 1.5 hours

WHAT YOU WILL NEED:

1. [ ] Vitals Equipment: Blood Pressure Monitor, Pulse Oximeter
2. [ ] Quiet Room
3. [ ] Computer
4. [ ] Piece of paper and pencil or pen
5. [ ] Zoom (video conference) link

SAMPLE

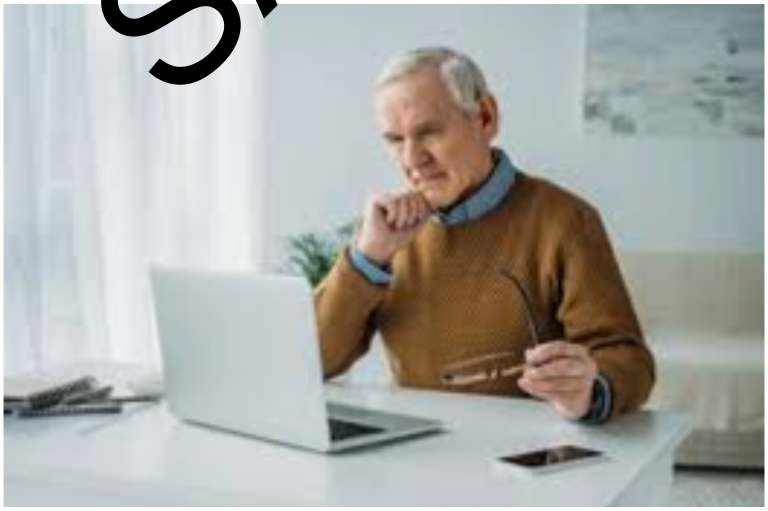

Section 5. Daily Exercise Sheet

You will join Zoom for your remote exercise session at your scheduled time.

Scheduled Date and Time:

Session #:

Visit time: 1 hour

WHAT YOU WILL NEED:

1. [ ] Vitals Equipment: Blood Pressure Monitor, Pulse Oximeter
2. [ ]Polar Heart Rate Monitor
3. [ ] Actigraph
4. [ ] Water
5. [ ] Connection to Zoom
6. [ ] Open space for exercise

SAMPLE

Vitals

PRE EXERCISE:

BLOOD PRESSURE:________________ HEART RATE:________________ OXYGEN SATURATION:________________

POST EXERCISE:

BLOOD PRESSURE:________________ HEART RATE:________________ OXYGEN SATURATION:________________

Reflections
