# Supplementary material for: Harnessing Neuroplasticity to Promote Brain Health in Aging Adults: Protocol for the MOVE-Cog Intervention Study
Source: JMIR Res Protoc. 2021 Nov 23;10(11):e33589. doi: 10.2196/33589 (PMC8663452; doi:10.2196/33589)
Supplement: Multimedia Appendix 2 [file resprot_v10i11e33589_app2.pdf]

CTSI KL2 Awards FY19 - Reviewer Evaluation Form

Dear Miami CTSI KL2 Reviewer,

Thank you for accepting to review this proposal.

Please submit your reviews by September 17, 2018.

If you have any question regarding this award, or the evaluation form, please contact Patricia Avissar at pavissar@med.miami.edu or 305-243-5085.

The RFA and Instructions are available on the CTSI website:
http://www.miamictsi.org/researchers/funding-opportunities/ctsi-k12-program-awards

Best regards,

The CTSI Team

Response was added on 09/14/2018 11:03am.

CONFLICT OF INTEREST

The following are considered potential conflicts for reviewing grant proposals:
• You have a financial and/or professional relationship with the applicant
• The funding decision on any application would benefit or affect you directly
• You feel there may be a perception of conflict
• You are a current collaborator, or have collaborated/published with the applicant in the last two years.

Note: You may review applications within the same department as long as none of the above conditions apply.

By checking the box below, you confirm that you do not have any conflict of interest in reviewing this proposal

☒ I confirm

SECTION 1: REVIEWER'S INFORMATION

SECTION 2: Miami CTSI KL2 AWARD APPLICANT INFORMATION

Applicant's Name (Last, First)

Gomes-Osman, Joyce

Department

Physical Therapy

Mentor(s)

Tatjana Rundek

Collaborator(s) on this project

David Loewenstein, Alvaro Pascual-Leone, Lawrence Cahalin

Proposal Title

Assessing cognitive improvements, brain neuroplasticity and the role of genetic factors after exercise in sedentary adults

SECTION 3: REVIEW

The focus of the KL2 award program of the Miami CTSI is to help outstanding early career investigators establish academic careers involving important translational, transformational research programs examining disease processes or outcomes related to health disparities.

Prior to proceed with this review, please read carefully the review criteria for the Miami CTSI KL2 Awards listed below:
• Compliance with all eligibility requirements (the administrative review is completed)
• Commitment and potential for a career in clinical and/or translational research.
• Commitment of the necessary resources and protected time from the primary department.
• Scholarship, as assessed from biosketch, letters of reference, and past academic performance.
• Evaluation of the proposed research project (scientific merit, potential clinical importance, scholar's ability to execute the research plan, available resources, likeliness of successful completion).
• Evaluation of the proposed mentored development plan, appropriateness and commitment of mentoring team and collaborators.
• Likelihood that the KL2 support will contribute to a successful K or R Award application (or equivalent).
• The impact of the research on improving the health of minority and underserved population.

The CTSI KL2 FY19 RFA and Instructions are attached here as one PDF.

[Attachment: "KL2 FY19 RFA & Instructions.pdf"]

A. Is this proposal a re-submission to the Miami CTSI KL2 Awards? (see Section 3 of the application)

⊗ No

B. SCORED REVIEW CRITERIA

1. Candidate

2. Research Plan

3. Mentoring Plan

4. Education Plan

The CTSI Pilot Awards Program utilizes the NIH 9-point rating scale (1=exceptional to 9=poor).

In assigning the scores, it is important that you consider the FULL RANGE of the rating scale . The scoring descriptors are displayed in each drop-down list.

1. CANDIDATE

CANDIDATE - STRENGTHS

The candidate is well qualified for this award. She has both a PhD and a clinical degree in Physical Therapy. She has a demonstrated commitment to a research career with 10 years of research experience beginning prior to her PhD studies and including at the Miami Project, as a Post Doc at Harvard and currently at the McKnight Center. She has 14 peer-reviewed publications with 8 as first author. She has also received internal research funding from McKnight Institute

CANDIDATE - WEAKNESSES

She has not received any type of external research funding as an investigator.

CANDIDATE - REVIEW SCORE - Use the FULL RANGE of the rating scale

⊗ 2- High Impact- OUTSTANDING- Extremely strong with negligible weaknesses

2. PROPOSED RESEARCH PLAN (Section 5 of the application)

- Significance: Is the research plan significant and does it address an important problem? Is the research plan consistent with the mission of the CTSI in terms of culturalized health sciences or to a disease issue of special concern in South Florida's unique racial/ethnic/sociocultural context? Is the research translational?

- Innovation: Is the project original and innovative?

- Approach: Are the conceptual, design, methods and analyses adequately developed, well-researched and appropriate to the aims of the proposed research project.

- Analysis Plan: Is the analysis plan on how to collect, analyze and interpret data adequate?

-Translational Plan: Does the candidate clearly describe the translational, clinical or public health impact of his/her research?

RESEARCH PLAN - STRENGTHS

Significance: Dr. Gomes-Osman's proposed research is focused on identifying the mechanisms underlying physical exercise interventions for age related cognitive decline. This is an important area of inquiry because, although there is evidence that physical exercise can improve cognition, much remains unknown concerning the relationships among changes in aerobic capacity, neuroplasticity and cognition and the possible role of genetic factors involved. The ultimate goal of this research is to develop and test personalized exercise protocols to promote cognitive brain health.
Innovation: The proposed project is innovative in exploring genetic differences that may modify exercise related cognitive improvements. The use of TMS to measure neuroplasticity to help identify mechanisms underlying exercise mediated cognitive improvement is also very innovative.
Approach: The proposed open trial, 8-week exercise intervention project appears well designed to address project aims. The investigator has proof of concept pilot data supporting the feasibility of the proposed study. The exercise intervention appears well designed. The proposed outcomes appropriately include neuroplasticity, cognitive performance, aerobic capacity and genetic information. The investigator proposes to recruit subjects from all of the major race-ethnic groups in South Florida.
Analysis Plan: The sample size calculation was based on change in executive function using an estimated effect size based on a recent meta-analysis and appears appropriate as do the proposed analyses.
Translational Plan: Appears appropriate.

RESEARCH PLAN - WEAKNESSES

Approach: The open trial design has the advantage of ensuring that all subjects receive the exercise intervention. However, the lack of a control group will make it difficult to determine to what degree TMS measured changes in neuroplasticity are due to fluctuations in the measure versus changes in aerobic capacity.
Analysis Plan: The proposed analysis plan does not include aerobic capacity. Factors such as variable levels of participation with the relatively demanding exercise intervention could produce variable levels of change in aerobic capacity. It would be worth exploring extent of improvement in aerobic capacity in relation to changes in neuroplasticity and cognition. It may be useful to include change in aerobic capacity when examining differences between genetic groups.
Translational Plan: The translation plan was not described in depth.

RESEARCH PLAN - SCORE - Use the FULL RANGE of the rating scale

⊗ 2- High Impact- OUTSTANDING- Extremely strong with negligible weaknesses

---

3. PROPOSED MENTORING PLAN (Section 6 of the application)

Please assess:
(1) the appropriateness of the research or teaching faculty mentors' qualifications in the area of this application
(2) the quality and extent of the mentors' proposed role in providing guidance and advice to the candidate
(3) the previous experience of the mentors in fostering the development of academic researchers
(4) the involvement of the mentor in the preparation of this proposal

---

MENTORING PLAN - STRENGTHS

Dr. Gomes-Osman's primary mentor is Tatjana Rundek, MD, PhD. Dr. Rundek is the Evelyn F. McKnight Chair for Learning and Memory in Aging and the Scientific Director of the Evelyn McKnight Brain Institute. She has expertise and experience in clinical and translational research as well as genetics and is extremely well qualified to mentor this particular applicant. Dr. Rundek and Dr. Gomes-Osman have an established relationship. The mentoring plan includes weekly meetings with Dr. Rundek focusing on research methodology and grant writing. Dr. Loewnstein will be a secondary mentor in the area of cognition and neuropsychological testing. Dr. Loewnstein is the Director of the Center for Cognitive Neuroscience and Aging. He is extremely well qualified to mentor Dr. Gomes-Osman in the area of age-related cognitive change and neuropsychological tool. The plan for regular meetings appears appropriate. Dr. Gomes-Osman also proposes to continue a mentorship relationship with Dr. Pascual-Leon of the Harvard Medical School. The letters of support from the mentors are very strong.

MENTORING PLAN - WEAKNESSES

Dr. Pascual-Leon is not on faculty at UM, however, the applicant has demonstrated ability to maintain a long distance mentorship relationship with him.

MENTORING PLAN - SCORE - Use the FULL RANGE of the rating scale

⊗ 1- High Impact- EXCEPTIONAL- Exceptionally strong with essentially no weaknesses

4. PROPOSED EDUCATION PLAN (Section 7 of the application)

Will this plan contribute to the scientific development of the scholar?
Please assess:
(1) the appropriateness of the content, the proposed duration of the career development plan to achieve scientific and academic independence for the scholar
(2) the usefulness of the plans for enhancing the academic skills as described in the career development plan.
(3) the likelihood that the career development plan will prepare the scholar to conduct research that will be informative/transformational concerning the biological, behavioral or sociocultural factors that influence susceptibility, progression or outcome of diseases that disproportionately affect South Florida's highly diverse racial/ethnic populations.
(4) the likelihood that the career development plan will prepare the individual for an academic career involving culturalized health sciences.

EDUCATION PLAN - STRENGTHS

The education plan appears very strong. It covers both years of the award and includes formal coursework in Public Health Sciences and Genetics as well as CTSI courses in research and grant-writing . The plan is very relevant to developing expertise in culturalized health science. The plan should also facilitate Dr. Gomes-Osman's long-term career goal of becoming an independent investigator and research leader.

EDUCATION PLAN - WEAKNESSES

None identified.

EDUCATION PLAN - SCORE - Use the FULL RANGE of the rating scale

⊗ 1- High Impact- EXCEPTIONAL- Exceptionally strong with essentially no weaknesses

C. ADDITIONAL REVIEW CRITERIA (NOT SCORED)
1. Addressing health disparities
2. Adequacy of Responsible Conduct of Research
3. Adequacy of Budget
4. Adequacy of Research Environment
5. Alignment with Medical School Strategic Plan

1. Does this proposal adequately address culturalized health or health disparities? Please provide a brief explanation to your response.

The applicant is a female, Latin-American researcher. Her research is focused on developing and testing personalized exercise protocols to promote cognitive brain health in participants recruited from all of the major race-ethnic groups in South Florida.

2. RESPONSIBLE CONDUCT OF RESEARCH (Section 7 of application)

Is the plan for providing formal and informal instruction in the bioethics, scientific integrity and the responsible conduct of research adequate?

⊗ Yes

3. BUDGET & BUDGET JUSTIFICATION (Section 8 of the application).

Is the budget acceptable as proposed?
NOTE: the proposed budget is a preliminary budget for $30K for research costs only. A detailed budget will be provided if the proposal is awarded.

⊗ Yes, the budget is acceptable as proposed.

---

4. ENVIRONMENT

Is the environment (academic, research and educational) described in the application conducive to the candidate achieving status as an independent investigator with capacity to prepare an NIH grant by the end of the funding period?
Is there adequate space and support for the proposed research project?

⊗ Yes

---

5. ALIGNMENT WITH MEDICAL SCHOOL STRATEGIC PLAN

Based on your review, is this project in alignment with the Medical School Research Strategic Plan? The link to the Medical School Research Strategic Plan visual overview is provided in the next field.

Please provide a short explanation to your response.

The applicant's research focus on interventions to improve cognitive health in aging individuals is well-aligned with the medical school's strategic plan under Neuroscience.

Miller School of Medicine Strategic Plan - PowerPoint slide

[Attachment: "MSOM Strategic Plan slide.pptx"]

D. SUMMARY SCORE

SUMMARY SCORE

Based on your overall review of this candidate's application, and your evaluation of the proposed research, education and mentoring plans in particular, please provide your Summary Score.
Note: the Summary Score does not have to be the average of the research, education and mentoring plans scores.

Use the FULL RANGE of the rating scale

⊗ 1- High Impact- EXCEPTIONAL- Exceptionally strong with essentially no weaknesses

E. ADDITIONAL COMMENTS (Optional)

Additional comments and recommendations to applicant

This applicant appears to be ideally suited for this program.

Have you joined the UM Internal Awards Reviewer Pool?

⊗ Yes
